# First report of coexistence of blaKPC-2 and blaNDM-1 in carbapenem-resistant clinical isolates of *Klebsiella aerogenes* in Brazil

**DOI:** 10.3389/fmicb.2024.1352851

**Published:** 2024-02-14

**Authors:** Saulo Henrique Rodrigues, Gustavo Dantas Nunes, Gabriela Guerrera Soares, Roumayne Lopes Ferreira, Marcelo Silva Folhas Damas, Pedro Mendes Laprega, Rebecca Elizabeth Shilling, Leslie Camelo Campos, Andrea Soares da Costa, Iran Malavazi, Anderson Ferreira da Cunha, Maria-Cristina da Silva Pranchevicius

**Affiliations:** ^1^Departamento de Genética e Evolução, Universidade Federal de São Carlos, São Carlos, São Paulo, Brazil; ^2^Laboratório Central de Saúde Pública do Tocantins, Palmas, Tocantins, Brazil

**Keywords:** *Klebsiella aerogenes*, whole-genome sequencing, resistance genes, mobile genetic elements, metabolic features, intensive care unit

## Abstract

*Klebsiella aerogenes* is an important opportunistic pathogen with the potential to develop resistance against last-line antibiotics, such as carbapenems, limiting the treatment options. Here, we investigated the antibiotic resistance profiles of 10 *K. aerogenes* strains isolated from patient samples in the intensive-care unit of a Brazilian tertiary hospital using conventional PCR and a comprehensive genomic characterization of a specific *K. aerogenes* strain (CRK317) carrying both the *bla*_KPC-2_ and *bla*_NDM-1_ genes simultaneously. All isolates were completely resistant to β-lactam antibiotics, including ertapenem, imipenem, and meropenem with differencing levels of resistance to aminoglycosides, quinolones, and tigecycline also observed. Half of the strains studied were classified as multidrug-resistant. The carbapenemase-producing isolates carried many genes of interest including: β-lactams (*bla*_NDM-1_, *bla*_KPC-2_, *bla*_TEM-1_, *bla*_CTX-M-1_ group, *bla*_OXA-1_ group and *bla*_SHVvariants_ in 20-80% of the strains), aminoglycoside resistance genes [*aac(6’)-Ib* and *aph*(*3’)-VI*, 70 and 80%], a fluoroquinolone resistance gene (*qnrS*, 80%), a sulfonamide resistance gene (*sul-2*, 80%) and a multidrug efflux system transporter (*mdtK*, 70%) while all strains carried the efflux pumps *Acr* (subunit A) and *tolC*. Moreover, we performed a comprehensive genomic characterization of a specific *K. aerogenes* strain (CRK317) carrying both the *bla*_KPC-2_ and *bla*_NDM-1_ genes simultaneously. The draft genome assembly of the CRK317 had a total length of 5,462,831 bp and a GC content of 54.8%. The chromosome was found to contain many essential genes. *In silico* analysis identified many genes associated with resistance phenotypes, including β-lactamases (*bla*_OXA-9_, *bla*_TEM-1_, *bla*_NDM-1_, *bla*_CTX-M-15_, *bla*_AmpC-1_, *bla*_AmpC-2_), the bleomycin resistance gene (*ble*_MBL_), an erythromycin resistance methylase (*ermC*), aminoglycoside-modifying enzymes [*aac(6’)*-*Ib*, *aadA/ant(3”)*-*Ia*, *aph(3’)-VI*], a sulfonamide resistance enzyme (*sul-2*), a chloramphenicol acetyltransferase (*catA-*like), a plasmid-mediated quinolone resistance protein (*qnrS1*), a glutathione transferase (*fosA*), PEtN transferases (*eptA*, *eptB*) and a glycosyltransferase (*arnT*). We also detected 22 genomic islands, eight families of insertion sequences, two putative integrative and conjugative elements with a type IV secretion system, and eight prophage regions. This suggests the significant involvement of these genetic structures in the dissemination of antibiotic resistance. The results of our study show that the emergence of carbapenemase-producing *K. aerogenes*, co-harboring *bla*_KPC-2_ and *bla*_NDM-1_, is a worrying phenomenon which highlights the importance of developing strategies to detect, prevent, and control the spread of these microorganisms.

## Introduction

*Klebsiella aerogenes*, previously identified as *Enterobacter aerogenes*, is a Gram-negative bacterium belonging to the *Enterobacteriaceae* family. It can be found in various environments including water, soil, air, and the human digestive system as a commensal organism. However, this bacterium is also a significant opportunistic pathogen that has been associated with hospital-acquired diseases such as pneumonia, meningitis, skin, soft tissue and urinary tract infections (Chen et al., 2015).

β-lactam antibiotics are one of the most commonly prescribed drug classes with numerous clinical uses. These antibiotics are sub-classed as penicillins (these being the most commonly prescribed), cephalosporins, cephamycins, monobactams, and carbapenems (Sta Ana et al., 2021). *Klebsiella aerogenes* is intrinsically resistant to ampicillin, amoxicillin, first-generation cephalosporins, and cefoxitin due to the expression of a constitutive AmpC β-lactamase. AmpC type lactamases are also known as extended spectrum β-lactamases (ESBLs) and these provide resistance against the majority of β-lactam antibiotics, such as extended-spectrum cephalosporins and monobactams, with the exception of carbapenems and cephamycins (Davin-Regli and Pages, 2015; Castanheira et al., 2021).

The presence of ESBLs in *K. aerogenes* strains has been well-documented, leading to the use of carbapenems as a last-resort treatment for serious infections caused by these pathogens. However, previous studies have shown a high prevalence of antibiotic resistance to cephalosporins and carbapenems in clinically relevant *K. aerogenes* strains worldwide (Ma et al., 2020). These infections pose a significant public health challenge due to the limited treatment options available and their elevated mortality rates (Mulani et al., 2019; Denissen et al., 2022).

The main resistant mechanism of carbapenemase-producing *K. aerogenes* is the production of carbapenemases, although other mechanisms have been proposed, including overproduction of β-lactamases, efflux pumps, porin deficiency, and changes in penicillin-binding proteins (Pan et al., 2021). Carbapenem resistance in clinical isolates of Carbapenem Resistant Enterobacterales (CRE) is predominantly caused by the presence of these carbapenemases, especially *Klebsiella pneumoniae* Carbapenamase (KPC) and New Delhi Metallo-β-lactamase (NDM). *bla*_KPC_ is commonly found on various plasmids like IncF-, IncI-, IncA/C-, IncX-, and IncR-type plasmids, while *bla*_NDM_ is mostly associated with IncX3-type plasmids. These plasmids are easily transferable and can promote the dissemination of *bla*_KPC_ and *bla*_NDM_ through horizontal gene transfer among diverse bacterial populations spreading antibiotic resistance (Yuan et al., 2023).

Although carbapenemases such as KPC, NDM, and Imipenemase (IMP) have been detected in *K. aerogenes*, there are limited studies demonstrating the simultaneous presence of *bla*_KPC_ and *bla*_NDM_ genes. Here, we conducted an in-depth analysis of genes related to the antimicrobial resistance and mobile genetic elements in carbapenemase-producing *K. aerogenes*, found in Brazilian hospitals, to understand its genomic diversity. To the best of our knowledge, this paper is the first to report the simultaneous presence of both *bla_KPC_* and *bla_NDM_* in *K. aerogenes* isolated from clinical samples in Brazil.

## Materials and methods

### Bacterial isolates

A total of 10 *K. aerogenes* were isolated from clinical specimens and devices in the ICU and neonatal intensive care unit (NICU) at a tertiary care of a government hospital in Palmas, Tocantins, Brazil, between January 2017 and May 2020. The *K. aerogenes* strains were initially identified by the hospital’s clinical microbiology laboratory before being forwarded to the Central Public Health Laboratory of the State of Tocantins (LACEN/TO) for species confirmation and drug susceptibility testing. LACEN is a healthcare facility under the Brazilian Ministry of Health that receives samples for antimicrobial resistance surveillance.

### Detection of antibiotic resistance and carbapenemase productions

Bacterial identification and determination of antibiotic susceptibility were carried out using the VITEK2 compact automated system (bioMerieux, Hazelwood, MO, USA). The susceptibility of the *K. aerogenes* isolates were tested against a panel of 16 antibiotics, which included ampicillin/sulbactam (SAM), piperacillin/tazobactam (TZP), cefuroxime sodium (CXM-S), cefuroxime axetil (CXM-AX), cefoxitin (FOX), ceftazidime (CAZ), ceftriaxone (CRO), cefepime (FEP), ertapenem (ETP), imipenem (IPM), meropenem (MEM), amikacin (AMK), gentamicin (GEN), ciprofloxacin (CIP), tigecycline (TGC), and colistin (CST). The findings were interpreted in accordance with the guidelines set forth by Clinical and Laboratory Standards Institute (CLSI, 2023). Phenotypic detection of carbapenemase production in *K. aerogenes* was carried out by modified Hodge test and ethylenediaminetetraacetic acid (EDTA) synergy tests under the CLSI guidelines (CLSI, 2023) as described elsewhere (Ferreira et al., 2019, 2020; Damas et al., 2022; Soares et al., 2023). *K. aerogenes* isolates were classified as multidrug-resistant (MDR) by non-susceptibility to at least one agent in three or more antimicrobial categories, as per the criteria established by Magiorakos et al. (2012). *K. aerogenes* are naturally resistant to ampicillin (AMP), amoxicillin/clavulanic acid (AMC), FOX, and cephalothin (CFL) due to the low production of the naturally induced cephalosporinase of Bush group 1 (class C) (Davin-Regli and Pages, 2015). Therefore, AMP and FOX were not included in the MDR classification (Magiorakos et al., 2012).

### DNA isolation

*Klebsiella aerogenes* strains were subcultured on Brain Heart Infusion (BHI) broth (Oxoid, United Kingdom) and incubated for 24 h at 37°C. Genomic DNA extraction was performed from an overnight culture using the Cellco Genomic DNA purification kit (Cellco Biotech., São Carlos, Brazil), according to the manufacturer’s instructions. The DNA was quantified using the NanoVue Plus instrument (GE Healthcare Life Sciences, Marlborough, MA, United States). The quality of the genomic DNA was examined through electrophoresis while the bacterial DNA concentration was determined using the Qubit® 3.0 fluorometer in combination with the Qubit® dsDNA Broad Range Assay Kit from Life Technologies (Carlsbad, CA, USA).

### Detection of antibiotic resistant genes

Polymerase chain reaction (PCR) was performed for the detection of resistance-related genes, such as ESBL-encoding genes (*bla*_TEM_, *bla*_SHV variants_, *bla*_OXA-1, 4 and 30_, *bla*_CTX-M-1 group_, *bla*_GES_, *bla*_PER-1 and 3_, *bla*_VEB-1 to 6_), carbapenemase genes (*bla*_KPC_, *bla*_OXA-48_, *bla*_IMP-1_, *bla*_VIM-2_, *bla*_NDM_, *bla*_SPM-1_, *bla*_GIM-1_, *bla*_SIM-1_), aminoglycosides [*armA*, *rmtB*, *aph(3′)-*VIa *(aphA6)*], tetracycline (*tetB*), sulfonamide (*sul-1*, *sul-2*), colistin resistance (*mcr-1*), plasmid mediated quinolone resistance (PMQR) gene [*aac(6’)-Ib-cr*, *qnrS1* and *qnrS2*], efflux pump (*acrAB*, *tol*C, and *mdtK*) genes. Amplicons were analyzed by gel electrophoresis in 1.0% agarose and visualized under ultraviolet (UV) light. Supplementary Table S1 provides information on amplicons length and PCR conditions.

One amplicon of each studied gene was purified using the Gel Band Purification Kit (Cellco Biotech., São Carlos, Brazil) and sequenced using the Sanger DNA sequencing method. The sequences were edited using Bioedit v7.0.5 (Hall, 1999), then compared with GenBank and Refseq sequences using BlastX tools: ACT53230.1 (*bla*_CTX-M-15_), QXU68638.1 (*bla*_TEM-1_), EKZ5222878.1 (*bla*_NDM_), SCZ84112.1 (*bla*_SHV-2_), WEA84669.1 (*bla*_KPC_), WP_240093217.1 (*bla*_OXA-1_), WP_047046709.1 (*tol*C), EFZ4507594.1 (*qnrS*), MCL7674773.1 (*acrA*), HBS1035150.1 (*aac-(6’)-Ib*), HEC1006964.1 (*aph(3’)-*VIa), QDB65114.1 (*sul*-*2*), and PLP19006.1 (*mdtK*). Subsequently, the nucleotide sequences of the genes were submitted to the GenBank database and assigned accession numbers: SRX22793090 (*sul-2*), SRX22793089 (*mdtK*), SRX22793063 (*qnrS*), SRX22793062 (*tolC*), SRX22793031 (*acrA*), SRX22789929 (*aph(3’)*-VIa), SRX22789927 (*aac-(6’)-Ib*), SRX22789878 (*bla*_SHV-2_), SRX22789871 (*bla*_OXA-1_), SRX22789802 (*bla*_NDM_), SRX22789553 (*bla*_KPC_), SRX22789857 (*bla*_TEM-1_), and SRX22789858 (*bla*_CTX-M-15_).

### Genome sequencing

The *K. aerogenes* CRKA317 was selected for whole genome sequencing (WGS). The Nextera XT DNA Library Prep Kit (Illumina, San Diego, California, United States) was utilized to conduct the library preparation using 1 ng of DNA as our material to sequence. A limited cycle polymerase chain reaction (PCR) program was employed to amplify the libraries introducing Index 1 (i7) adapters, Index 2 (i5) adapters, and the requisite sequences for generating sequencing clusters. The amplified library was purified using 0.6 x Agencourt AMPure XP beads (Beckman Coulter, Brea, California, USA). The quality of the library and the size of fragmented DNA was evaluated on a 1.5% electrophoresis agarose gel and quantified using a fluorometric method involving the Qubit® 3.0 instrument and the Qubit® dsDNA Broad Range Assay Kit (Life Technologies, Carlsbad, California, United States). The resulting library concentrations were subsequently normalized to 4 nM using a standard dilution method. The libraries were then combined, denatured with 0.2 N sodium hydroxide (NaOH), and diluted to attain a final concentration of 1.8 pM. To ensure the run’s accuracy and control, a PhiX control was added to achieve a final concentration of 1.5 pM. The sequencing run involved a paired-end run comprising 75 cycles for each read (2 × 75), plus up to eight cycles for two index reads.

### Genome assembly, annotation and prediction of orthologous group

Initially, FastQC v.0.12.0^1^ was used to check the raw reads quality. The raw reads were filtered by quality, length, and adapter regions using Trim Galore! v.0.6.10.^2^ The genome assembly was made with SPAdes 3.2 (Bankevich et al., 2012) and SSPACE (Boetzer et al., 2011), using “careful” and “cov-cutoff auto” as settings. Contigs with less than 200 bp were discarded. PlasmidFinder 2.13^3^ (Carattoli et al., 2014) and PlasmidSPAdes (Antipov et al., 2016) were used to plasmid detection and assembly attempts. QUAST v5.0.2 (Gurevich et al., 2013) were used to access the general statistics of assembled genome. The circular genome was built using Proksee^4^ (Grant et al., 2023). For the annotations of genome, both Prokka v.1.14.5 (Seemann, 2014) and Rapid Annotation using Subsytems Technology (RAST)^5^ (Aziz et al., 2008) servers were used. The completeness of the assembled genome was assessed using the BUSCO program (Simão et al., 2015).

The Clusters of Orthologous Group (COG) were annotated and distributed in categories using eggNOG-mapper v2^6^ (Cantalapiedra et al., 2021). Kyoto Encyclopedia of Genes and Genomes (KEGG) was used to determinate Gene Ontology (GO)^7^ (Kanehisa et al., 2016).

### Phylogenetic inferences using 16S rRNA gene sequences, ANI, dDDH, and TYGS

Our 16S rRNA gene sequence from our genome annotation was used in the analysis with another 36 16S rRNA reference sequences of *Klebsiella* genus obtained from the GenBank database (Supplementary Table S2). The 16S rRNA analysis was performed using *Escherichia coli* as its outgroup. Nucleotide sequences were aligned using the online software MAFFT^8^ (Katoh and Standley, 2013). JModelTest v2.1.10 (Posada, 2008) was used to estimate the best-fitting nucleotide substitution model and PhyML v3.0 (Guindon et al., 2009) to construct a maximum likelihood (ML) phylogenetic tree. Branches was supported by bootstrap analysis of 1,000 replicates.

The OrthoANI v0.93.1 tool (Yoon et al., 2017) was used to calculate the Average Nucleotide Identity (ANI) between our genome and another 18 reference and uncharacterized complete *Klebsiella* genus genomes (Supplementary Table S3). For the *in silico* calculation of digital DNA–DNA Hybridization (dDDH), Genome to Genome Distance Calculator (GGDC 3.0)^9^ (Meier-Kolthoff et al., 2013) was used with the same genomes. A heatmap with the results from OrthoANI and dDDH was constructed using CIMminer.^10^ To reinforce our phylogenetic inference, the Type (Strain) Genome Server (TYGS)^11^ was performed using all strains from the server database (Meier-Kolthoff et al., 2022).

### Comparative pan-genome analysis of *Klebsiella aerogenes* strains

The online pipeline REALPHY^12^ (Bertels et al., 2014) was used to build a whole-genome sequence-based phylogenetic tree, using the 26 complete genomes of clinical strains of *K. aerogenes* available in NCBI (Supplementary Table S4). The result showed the closest *Klebsiella* species to our strain. These species were used in Orthovenn2 web server^13^ (Xu et al., 2019) to compare orthologous gene clusters using whole-genome sequence. Furthermore, Bacterial Pangenome Analysis Pipeline (BPGA) v.1.3 (Chaudhari et al., 2016) was performed against the Kyoto Encyclopedia Genomics and Genes Database (KEGG) to predict the core, accessory and unique genes as well as their functional distribution. REALPHY, Orthovenn2 and BPGA were used in default settings.

### Characterization of resistance

The annotation of antibiotic resistance genes, efflux pumps and porins was made by CARD online^14^ (Alcock et al., 2019), ResFinder 4.4.2^15^ (Bortolaia et al., 2020), ABRicate^16^ (Afgan et al., 2018), BlastKOALA^17^ (Kanehisa et al., 2016) and CARD and ARG-ANNOT (Gupta et al., 2014) databases. The parameters used for databases were 1E-5 e-value, ≥ 70% of identity and ≥90% coverage cut-off.

Known mutations in *gyrA*, *gyrB*, and *parC*, that are responsible for quinolone resistance, were investigated using BLASTp comparison. Furthermore, even though *K. aerogenes* CRKA317 is not resistance to colistin, mutations in *phoP* and *phoQ* were also evaluated to investigate polymyxin resistance. For the alignment we used the following sequences: *gyrA* (*Klebsiella* [multispecies]: WP_004201688.1), *gyrB* (*Klebsiella* [multispecies]: WP_004173845.1), *parC* (*Klebsiella* [multispecies]: WP_004181324.1), *phoP* (*Klebsiella* [multispecies]: WP_025714403.1) and *phoQ* (*Klebsiella* [multispecies]: WP_045393745.1).

### Genomic islands and mobile genetic elements

The presence of Genomic Islands (GIs) was investigated with IslandViewer 4 webserver^18^ (Bertelli et al., 2017), using *K. aerogenes* isolate 57 as the reference strain. Integrons, transposons and insertion sequences were evaluated using Integron Finder (Afgan et al., 2018), TnCentral^19^ (Ross et al., 2021) and ISfinder^20^ (Siguier, 2006), respectively. MGEfinder^21^ (Durrant et al., 2020) was used to understand the relation between resistance genes with mobile genetic elements. The webserver ICEfinder^22^ (Liu et al., 2019) was used to identify Integrative and Conjugative Elements (ICE). Sequences of Clustered Regularly Interspaced Short Palindromic Repeats (CRISPR) were searched using CRISPRCas Finder^23^ (Couvin et al., 2018). To identify and annotate prophage sequences in genome, the PHASTER webserver^24^ (Arndt et al., 2016) was used. The Phigaro v.2.3.0 pipeline (Starikova et al., 2020) was used to indicate the possible phage family.

### Genome accession number

Raw reads were submitted to Sequence Reads Archives,^25^ with submission number JAXIVA000000000. The draft genome is available at GenBank BioProject accession PRJNA1047945.

## Results

### Antimicrobial susceptibility, detection of resistance-related genes

A total of 10 non-repetitive clinical isolates of *K. aerogenes* were isolated from rectal swabs, (40%, *n* = 4), urine (30%, *n* = 3), tracheal aspirate (10%, *n* = 1), blood (10%, *n* = 1) and catheter tips (10%, *n* = 1) from adult patients admitted to the intensive care unit (ICU) of a tertiary hospital located in Brazil. All isolates were resistant to the β-lactam antibiotics tested, including SAM, TZP, CXM-S, CXM-AX, FOX, CAZ, CRO, FEP, ETP, IPM, and MEM. MDR was observed in 50% (*n* = 5) of the strains, and the most common MDR profiles were related to β-lactam-aminoglycosides-quinolone (20%, *n* = 2), β-lactam-quinolone-glicylcycline (20%, *n* = 2), and β-lactam-aminoglycosides-quinolone-glicylcycline (10%, *n* = 1). On the other hand, 80% of the isolates were susceptible to GEN, 80% to AMK, 70% to TGC, 50% to CIP, and 100% to CST. General data and susceptibility profiles of all clinical carbapenem-resistant *K. aerogenes* (CRKA) isolates are showed in Table 1 and Supplementary Figure S1.

**Table 1:** Antimicrobial resistance of *K. aerogenes* isolates and presence of genes coding for resistance, and efflux pumps.

Strains	Source of infection	Antibiotic resistance	MDR	Genes associated with drug resistance												
				β-lactams						Aminoglycosides		Quinolone	Sulfonamide	Multidrug efflux pump		
				*bla*_KPC-2_	*bla_NDM-1_*	*bla_TEM-1_*	*bla_oxa1, 4, 30_*	*bla*_SHV variants_	*bla*_CTX-M-1 group_	*aac(6’)-Ib*	*aph(3’)-*VIa	*qnrS (qnrS1, S2)*	*sul-2*	*acrA*	*tolC*	*mdtK*
CRKA315	Rectal swab	SAM, TZP, CXM, CXM-S, FOX, CAZ, CRO, FEP, ETP, IPM, MEM	No	+	–	–	–	+	+	+	+	+	+	+	+	+
CRKA316	Rectal swab	SAM, TZP, CXM, CXM-S, FOX, CAZ, CRO, FEP, ETP, IPM, MEM	No	+	+	+	–	–	+	+	+	+	+	+	+	–
CRKA454	Rectal swab	SAM, TZP, CXM, CXM-S, FOX, CAZ, CRO, FEP, ETP, IPM, MEM, CIP, TGC	Yes	–	+	+	+	–	–	+	+	+	+	+	+	+
CRKA534	Rectal swab	SAM, TZP, CXM, CXM-S, FOX, CAZ, CRO, FEP, ETP, IPM, MEM, CIP, TGC	Yes	+	+	+	–	–	+	+	+	+	+	+	+	+
*CRKA317	Urine	SAM, TZP, CXM, CXM-S, FOX, CAZ, CRO, FEP, ETP, IPM, MEM, AMK, CIP, TGC	Yes	+	+	+	+	-	+	+	+	+	+	+	+	+
CRKA459	Urine	SAM, TZP, CXM, CXM-S, FOX, CAZ, CRO, FEP, ETP, IPM, MEM	No	+	+	–	–	–	–	–	+	+	+	+	+	–
CRKA538	Urine	SAM, TZP, CXM, CXM-S, FOX, CAZ, CRO, FEP, ETP, IPM, MEM, GEN, CIP	Yes	+	+	+	+	–	+	+	+	+	+	+	+	+
CRKA532	Tracheal aspirate	SAM, TZP, CXM, CXM-S, FOX, CAZ, CRO, FEP, ETP, IPM, MEM	No	–	+	+	+	–	–	+	–	–	–	+	+	+
CRKA211	Blood	SAM, TZP, CXM, CXM-S, FOX, CAZ, CRO, FEP, ETP, IPM, MEM, AMK, CIP	Yes	–	+	+	–	–	–	+	–	+	+	+	+	+
CRKA495	Catheter tip	SAM, TZP, CXM, CXM-S, FOX, CAZ, CRO, FEP, ETP, IPM, MEM	No	+	–	+	–	+	–	–	–	–	–	+	+	–
		**Genes present (%)**		**70**	**80**	**80**	**40**	**20**	**50**	**80**	**70**	**80**	**80**	**100**	**100**	**70**

Of the 10 carbapenemase-producing *K. aerogenes* isolates, the *bla*_NDM-1_ gene was detected in 8 isolates (80%), followed by *bla*_KPC-2_ in 7 isolates (70%). Whereas the concomitant presence of *bla*_KPC-2_ with *bla*_NDM-1_ gene was detected in 5 isolates (50%). In addition, the *bla*_TEM-1_ (80%, *n* = 8) was the most common ESBL-encoding gene among *K. aerogenes* investigated, followed by *bla*_CTX-M1-group_ (50%, *n* = 5), *bla*_OXA-1,4, and 30_ (40%, *n* = 4), and *bla*_SHV variants_ (20%, *n* = 2) (Table 1 and Supplementary Figure S1).

Regarding the genes that provide resistance to aminoglycosides, 7 isolates (70%) carried the *aph(3′)-VI* (*aphA6*) and 8 strains (80%) carried the *aac(6’)-Ib* gene. Eight strains (80%) harbored *qnrS* (*qnrS1* and/or *qnrS2*), capable of causing resistance fluoroquinolones antibiotics. The sulfonamide resistance gene (*sul-2*) gene was present in 8 isolates (80%). All the CRKA isolates we investigated had genes related to efflux pumps *acrA* and *tolC*. The *mdtK* gene, which is a multidrug efflux system transporter, was present in 7 strains (70%) (Table 1 and Supplementary Figure S1). The genes related to antibiotic resistance *bla*_OXA-48_; *bla*_SPM-1_; *bla*_IMP-1_; *bla*_VIM-2_; *bla*_SIM-1_; *bla*_GIM-1_, *bla*_GES-1, 9,11_; *bla*_PER-1, 3_; *bla*_VEB-1 to 6_, *mcr-1*, *sul-1*, *aac(6′)-Ib-cr*, *armA*, *rmtB* and *tetB* were not found in CRKA isolates.

### Classes of antibiotics

β-lactams: SAM (ampicillin-sulbactam), TZP (piperacillin-tazobactam), CXM-S (cefuroxime sodium), CXM (cefuroxime axetil), FOX (cefoxitin), CAZ (ceftazidime), CRO (ceftriaxone), FEP (cefepime), ETP (ertapenem), IPM (imipenem), MEM (meropenem); aminoglycosides: GEN (gentamicin) and AMK (amikacin); quinolones: CIP. (ciprofloxacin); glycylcycline: TGC (tigecycline) and polymyxin: CST (colistin). MDR (multidrug-resistant) = resistance to at least one agent in three or more antibiotic categories. * Whole-genome sequencing was performed on CRKA317. +, the tested gene was detected by PCR and Sanger sequencing; –, the tested gene was not detected.

### Genome and functional annotation

**Figure 1:**
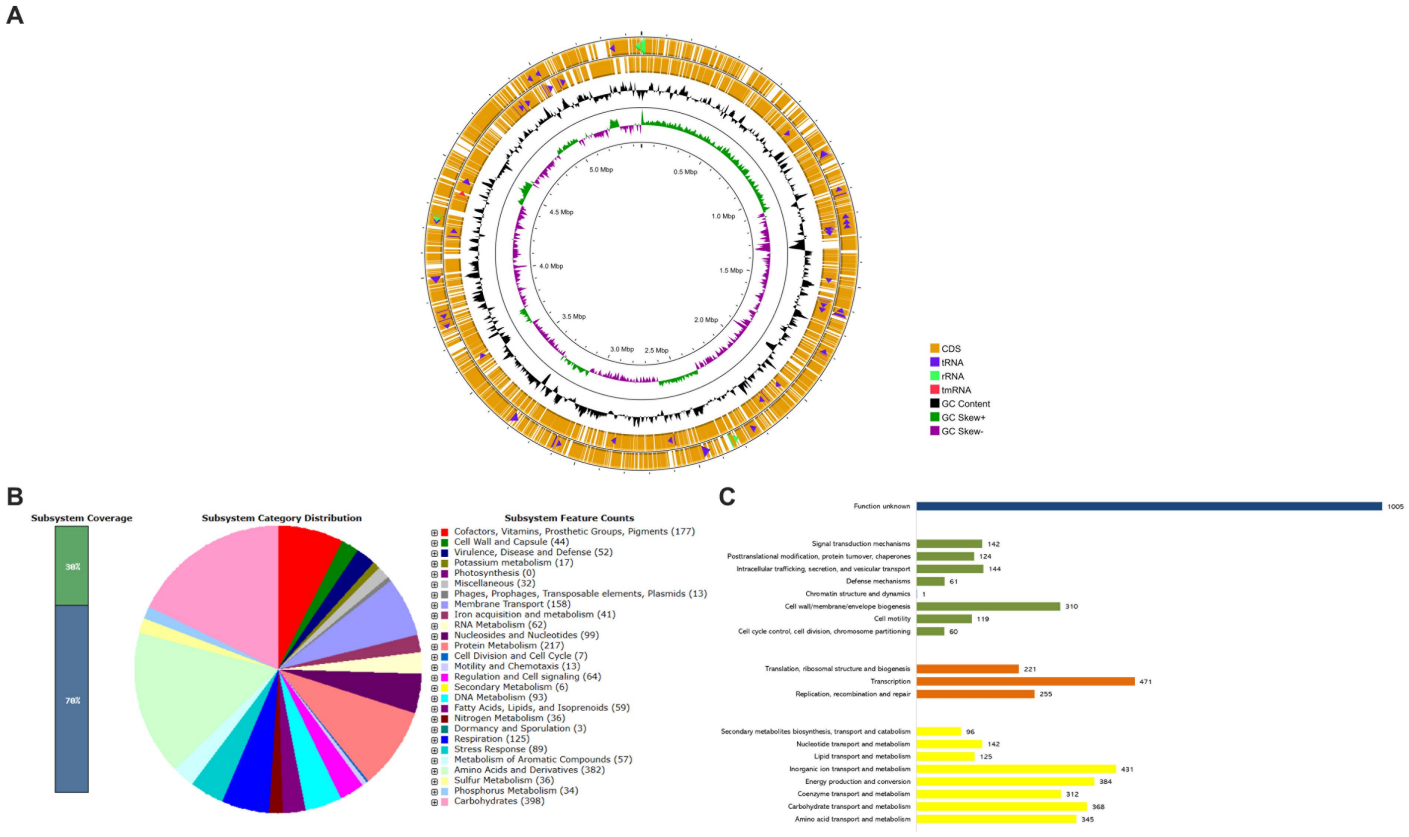
Whole-Genome Sequencing and functional annotations of *K. aerogenes* CRKA317. **(A)** The circular diagram consists of four circles, numbered from 1 (outer) to 4 (inner). The outer two orange circles represent the coding sequence (CDS), the transfer RNA (tRNA) is indicated by a purple arrowhead, the ribosomal RNA (rRNA) is shown with a green arrowhead, and the transfer-messenger RNA (tmRNA) is denoted by a red arrowhead. The third, black circle represents the GC content. The fourth circle displays the GC skew curve indicating a positive skew in green and negative skew in violet. **(B)** The bar indicates the percentage of subsystem coverage, with the green segment representing the proportion of proteins included. The pie chart presents an overview of the distribution of various categories within these subsystems. **(C)** COG classification in categories: metabolism (yellow bars), information processing and storage (orange bars), cellular processes and signaling (green bars), and unknown function (blue bar).

Given the existence of both the *bla*_KPC-2_ and *bla*_NDM-1_ genes in CRK317, as well as the strain’s resistance to a broad spectrum of antibiotics (except for gentamicin and colistin), whole genome sequencing (WGS) was employed to obtain comprehensive genomic data from the *K. aerogenes* CRKA317. The draft genome of CRKA317 comprised one circular chromosome, which is 5,462,831 bp in size, with an average GC content of 54.8%. The annotation of the bacterial genome predicted a total of 51 contigs, 5,403 coding sequences and 5,374 genes that covered 88.88% of genome. Of the 65 RNA genes predicted, 5 were rRNAs, 59 were tRNAs and one was a transfer-messenger RNA (tmRNA) (Supplementary Table S5 and Figure 1A). The assembly of plasmids was unsuccessful, but fragments of IncFIB (pQil), IncC, and IncFII (K) plasmids were detected.

According to the RAST analysis, the genome of *K. aerogenes* CRKA317 is composed of 398 subsystems that can be categorized into 27 distinct categories (Figure 1B). The six most significant categories included “carbohydrates” with a total of 398 genes, followed by “amino acids and derivatives” (382 genes), “protein metabolism” (217 genes), “cofactors, vitamins, prosthetic groups, pigments” (177 genes), “membrane transport” (158 genes), and “respiration” (125 genes). In the specific category of “virulence, disease and defense,” (52 genes) there were 32 genes related to resistance against antibiotics and toxic compounds; such as β-lactamase enzymes (one gene), fluoroquinolone resistance (two genes), fosfomycin resistance (one gene), copper homeostasis (11 genes), copper homeostasis: cooper tolerance (ten genes), cobalt-zinc-cadmium resistance (four genes), zinc resistance (two genes), adaptation to d-cysteine (one gene); Furthermore, we found 14 genes associated with invasion and intracellular resistance, four genes linked to adhesion, and two genes related to bacteriocins, ribosomally synthetized antibacterial peptides.

The analysis of protein-coding genes resulted in a total of 5,116 genes distributed across different functional categories within the Cluster of Orthologous Groups. The largest proportion of known protein coding genes was related to “transcription” (471; 9.21%), followed by categories such as “inorganic ion transport and metabolism” (431; 8.42%), “energy production and conversion” (384; 7.50%), “carbohydrate transport and metabolism” (368; 7.19%), and “amino acid transport and metabolism” (345; 6.74%). There were also gene associations with defense mechanisms (61; 1.19%) and a significant portion classified as having unknown functions (1,005; 19.64%) (Figure 1C).

### Phylogenetic analysis and genome similarity among representative *Klebsiella* species

**Figure 2:**
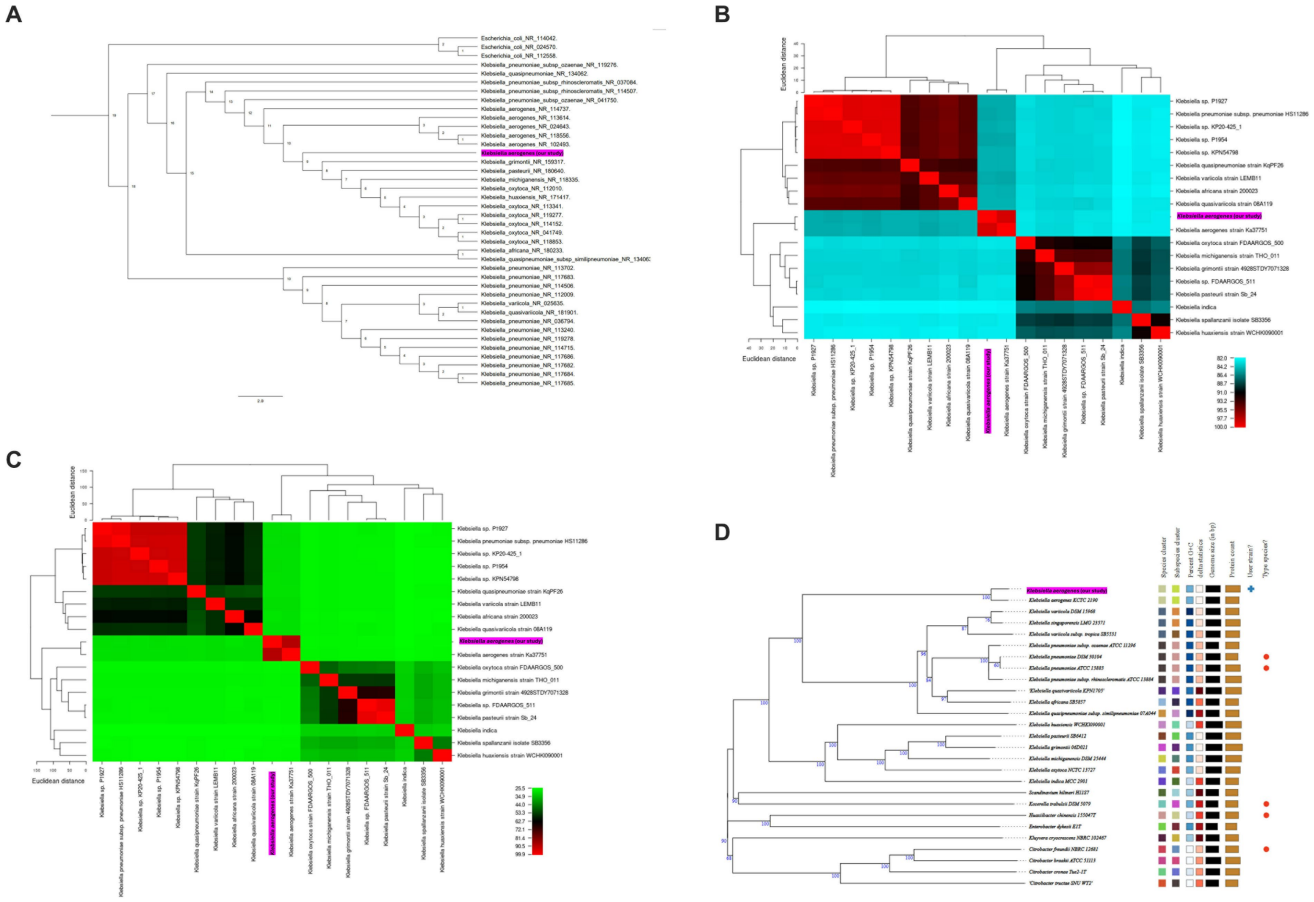
**(A)** Phylogenetic tree based on 16S rRNA gene sequences, which shows the relationship between *K. aerogenes* CRKA317 and other. The number next to the node represents the age value, and the scale bar indicates 2.0 substitutions per nucleotide position. **(B,C)** display heat maps of average nucleotide identity (ANI, **B**) and *in silico* DNA–DNA hybridization (DDH, **C**) respectively, which compare *K. aerogenes* CRKA317 to *Klebsiella* species. **(D)** Phylogenomic tree based on TYGS. The numbers above the branches represent GBDP pseudo-bootstrap support values >60% of 100 replications, with an average branch support of 86.2%. The tree was rooted at the midpoint. Our strain is highlighted in pink.

To gain insights into the evolutionary placement of *K. aerogenes* CRKA317, a phylogenetic tree was generated using 16S rRNA gene sequences from 36 reference sequences of *Klebsiella* species available at NCBI.^26^ Our findings indicated that CRKA317 was not closely related to *K. aerogenes* (Figure 2A). The use of 16S rRNA gene for species identification presents significant challenges in interpretation because of its hypervariable domains (Koroiva and Santana, 2022). Nevertheless, this approach allowed for an assessment of its relationship in the broader context of the genus.

Next, species validation and genomic similarity were assessed through *in silico* ANI, DDH and TYGS analysis. Our next step was a comparative analysis of 13 reference sequences of *Klebsiella* species, including 5 clinical isolates of *Klebsiella* spp. and our *K. aerogenes* CRKA317. ANI analysis revealed high similarity between *K. aerogenes* CRKA317 and *K. aerogenes* (Ka37751; GCA_007632255.1), with a close match of approximately 98.52% (Figure 2B). The genetic relatedness between these two strains was also confirmed with a DDH value of 88.90% (Figure 2C). The TYGS-based results showed that *K. aerogenes* CRKA317 is most closely related to *K. aerogenes* KCTC 2190, with dDDH values of 89%, also positioning CKA317 as a *K. aerogenes* (Figure 2D).

### Phylogenomic analysis of *Klebsiella aerogenes* strains

**Figure 3:**
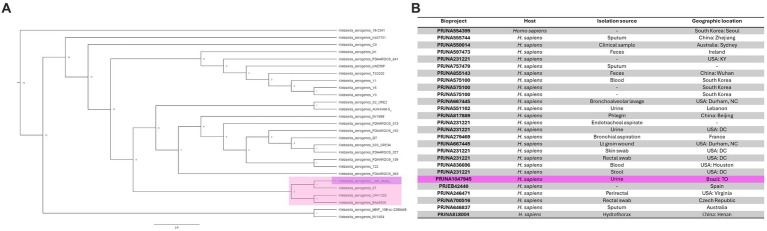
Phylogenomic analysis of *K. aerogenes* strains. **(A)** Phylogenetic tree showing the similarity between CRKA317 and other 26 selected strains of *K. aerogenes*. **(B)** Genome Assembly and Annotation report of *K. aerogenes* strains. Our strain is highlighted in pink.

Next, we determined the genetic similarity between *K. aerogenes* CRKA317 and 26 genomes of *K. aerogenes* obtained from the NCBI database. Our findings indicated that our *K. aerogenes* CRKA317 strain is more closely related to *K. aerogenes* 57, *K. aerogenes* CAVI1320, and *K. aerogenes* EA46506, which are forming a monophyletic clade (Figure 3A). Although the isolation source for the closest strain (*K. aerogenes* 57) was not specified in the NCBI website, the other two strains were isolated from clinical samples (Figure 3B).

### Comparative genomic analysis of four *Klebsiella aerogenes* strains

**Figure 4:**
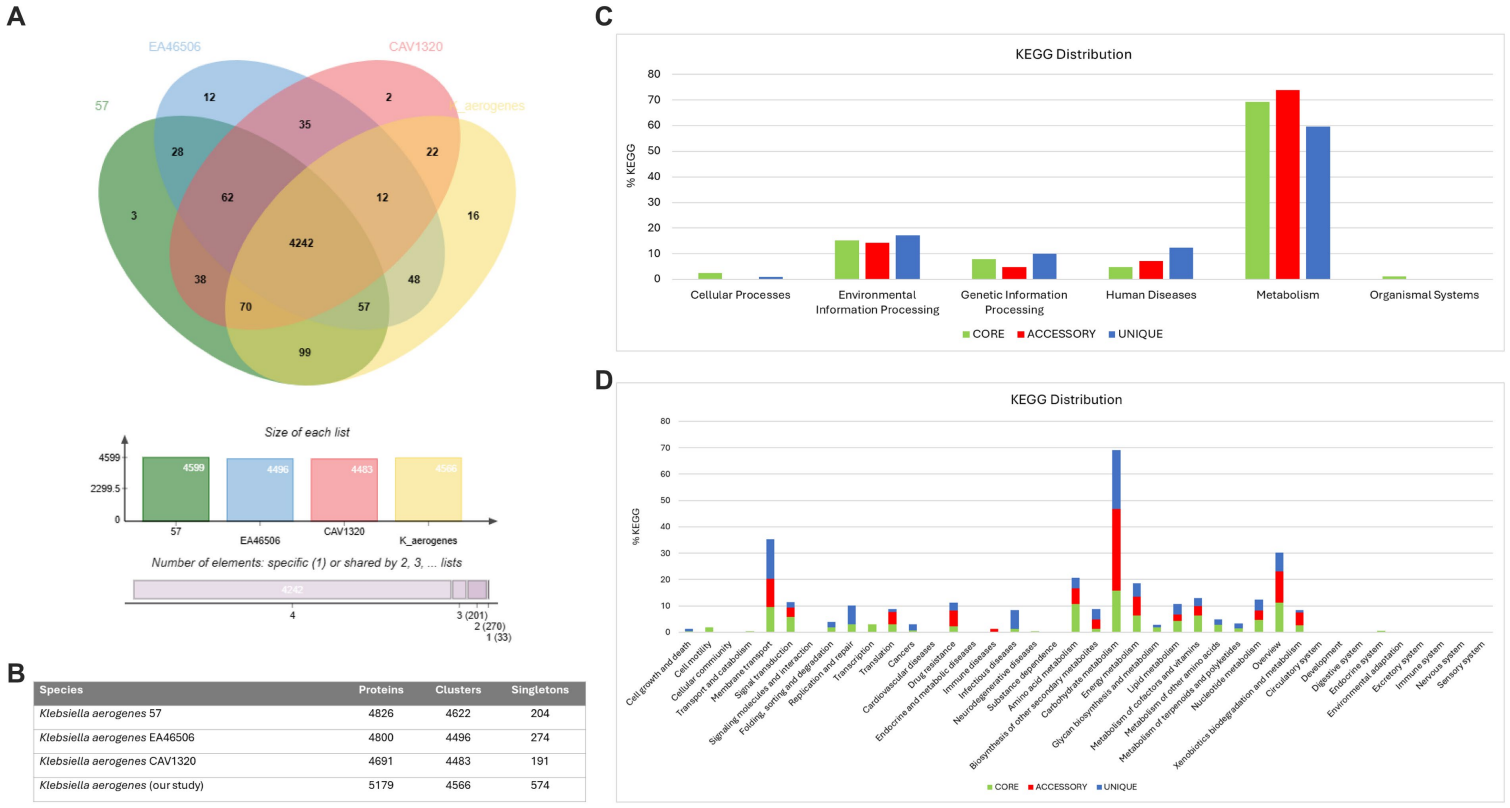
Comparative genomic analysis. **(A)** Venn diagram and bar chart showing the numbers of unique and shared orthologous genes present in most closely related strains of *K. aerogenes*. **(B)** Number of proteins, clusters and singletons. **(C)** KEGG pathway classification in core, accessory and unique genomes. **(D)** Distribution of KEGG pathway classification.

We also conducted a comparative analysis of the predicted gene numbers in three closely related strains of *K. aerogenes* with our *K. aerogenes* CRKA317. This allowed us to identify both common genes shared across these strains, as well as those that were unique to each individual strain. Our data showed the four *K. aerogenes* strains shared 4,242 genes, and *K. aerogenes* CRKA317 was found to contain 16 strain-specific gene clusters which were associated to metal ion transport including response to cadmium ion, and copper ion transport (Figure 4A). The *K. aerogenes* CRKA317 showed a higher number of singleton genes (*n* = 574) compared to other *K. aerogenes* strains (Figure 4B), including genes associated with resistance such as *bla*_NDM-1_, *aad*A/*ant*(3”)-Ia, *aph*(3’)-VI, and *qnr*S1.

The analysis of KEGG functional distributions in the 4 strains (*K. aerogenes* 57, *K. aerogenes* CAVI1320, and *K. aerogenes* EA46506) showed that the majority of genes were associated with the core genomes (94.16%; *n* = 2,950), followed by unique genomes (3.16%; *n* = 99), and accessory genomes (2.7%; *n* = 84) (Figure 4C). The genes were associated mainly with “metabolism” and were highly abundant in the accessory genome (73.91%; *n* = 62), followed by the core (69.29%; *n* = 2047), and unique (59.59%, *n* = 59) genomes (Figure 4C). Out of the total number of genes linked to human diseases (*n* = 158), 12.3% were found in the unique gene clusters, while 7.14% were present in accessory gene clusters and 4.73% was assigned to core gene clusters (Figure 4C).

Analysis of the annotations for all core genes revealed that a majority were associated with “carbohydrate metabolism” (15.83%), “overview” (11.2%), and “amino acid metabolism” (10.68%). Within the unique genome, significant proportions of genes were identified as belonging to categories such as “carbohydrate metabolism” (22.22%), “membrane transport” (15.15%), and “overview,” “infectious disease” and “replication and repair” with similar values (7.07%). In the accessory genome, most genes were categorized as “carbohydrate metabolism” (30.95%), “overview” (11.9%) and “membrane transport” (10.7%) (Figure 4D). Notably, we observed the presence of genes related to β-lactam resistance (0.61%, *n* = 18), vancomycin resistance (0.27%, *n* = 8), and cationic antimicrobial peptide resistance (1.35%, *n* = 40) within the core gene clusters specifically linked to drug resistance (Figure 4D).

### Resistome of *Klebsiella aerogenes* CRKA317

Resistome analysis using WGS revealed that *K. aerogenes* CRKA317 harbored dozens of antibiotic resistance-associated genes, including genes coding for β-lactamases (*bla*_OXA-9_, *bla*_TEM-1_, *bla*_NDM-1_, *bla*_CTX-M-15_, *bla*_AmpC-1_, *bla*_AmpC-2_); aminoglycoside-modifying enzymes [*aac(6’)-Ib*, *aadA/ant(3”)-Ia*, *aph(3’)-VI*], a chloramphenicol acetyltransferase (*catA-like* chloramphenicol resistance), an erythromycin resistance methylase (*ermC*), a plasmid-mediated quinolone resistance protein (*qnrS1*), a sulfonamide resistance enzyme (*sul-2*), a glutathione transferase (*fosA*: fosfomycin resistance), PEtN transferases (*eptA* and *eptB*: resistance to peptide antibiotic), a glycosyltransferase (*arnT*: resistance to peptide antibiotic) (Table 2). We also found the *ble*_MBL_ gene that encodes a bleomycin resistance protein (BRP). Although the majority of resistance genes identified through sequencing were consistent with those detected in whole genome sequencing (Table 1), *bla*_KPC-2_ was only detected through Polymerase Chain Reaction-based amplification and sequencing, but not predicted from incomplete genomic sequence of *K. aerogenes* CRKA317.

**Table 2:** Identification of the antibiotic resistance genes in the genome of *K. aerogenes* CRKA317.

Phenotypic antibiotic class	Phenotypic antibiotic resistance (Vitek 2)	Reference sequence (NCBI)	Putative resistance genes	Resistance gene/protein, mechanism function	Size (aa)	Coverage	aa identity (%)	Resistance gene characterization
**β-lactam**	Ampicillin-sulbactam, Piperacillin-tazobactam, Cefuroxime sodium Cefuroxime axetil, Cefoxitin, Ceftazidime, Ceftriaxone, Cefepime, Ertapenem, Imipenem, Meropenem	WP_282563773.1	*bla*_OXA-9_	Class D β-lactamase OXA	284	100	100	CARD, ABRicate, ResFinder, Arg-Annot, KEGG, Prokka
		WP_000027057.1	*bla*_TEM-1_	Class A broad-spectrum β-lactamase TEM	286	100	100	CARD, ABRicate, ResFinder, Arg-Annot, KEGG, Prokka
		WP_004201164.1	*bla*_NDM-1_	Class B broad-spectrum β-lactamases NDM	270	100	100	CARD, ABRicate, ResFinder, Arg-Annot, KEGG, Prokka
		WP_000239590.1	*bla*_CTX-M-15_	Class A extended-spectrum β-lactamases CTX-M	291	100	100	CARD, ABRicate, ResFinder, Arg-Annot, KEGG, Prokka
		KAA0468326.1	*bla*_AmpC-1_	Class C β-lactamase	382	100	100	KEGG, Prokka, BLAST
		OUE80029.1	*bla*_AmpC-2_	Class C β-lactamase	386	100	100	KEGG, Prokka, BLAST
**Aminoglycosides**	Amikacin	WP_004152783.1	*aac(6’)-Ib*	Aminoglycoside 6’ *N*-acetyltransferase	201	100	100	CARD, ABRicate, ResFinder, Arg-Annot, KEGG, Prokka
		WP_247187715.1	*aadA/ANT(3”)-Ia*	Aminoglycoside nucleotidyltransferase	262	82.51	100	CARD, ABRicate, ResFinder, Arg-Annot, KEGG, Prokka
		WP_014386410.1	*aph(3’)-VI*	Aminoglycoside 3’-*O* Phosphotransferase enzymes	259	100	100	CARD, ABRicate, ResFinder, Arg-Annot, KEGG, Prokka
**Quinolones**	Ciprofloxacin	WP_001516695.1	*qnrS1*	Plasmid-mediated quinolone resistance	218	100	100	CARD, ABRicate, ResFinder, Arg-Annot, KEGG, Prokka
**Sulfonamide**	NT	WP_011270145.1	*sul-2*	Sulfonamide resistant dihydropteroate synthase	283	100	100	CARD, ABRicate, ResFinder, Arg-Annot, KEGG, Prokka
**Fosfomycin**	NT	WP_015704268.1	*fosA*	Glutathione S-transferase	139	90.6	100	CARD, ABRicate, ResFinder, Arg-Annot, KEGG, Prokka
**Macrolides**	NT	WP_107318659.1	*ermC*	23S ribosomal RNA methyltransferase	289	100	100	KEGG, Prokka, BLAST
**Phenicol**	NT	WP_074165951.1	*catA-like*	Chloramphenicol acetyltransferase	221	100	100	KEGG, Prokka, BLAST
**Bleomycin**	NT	WP_004201167.1	*bleMBL*	Bleomycin binding protein	121	100	100	CARD, Prokka, BLAST
**Peptide antibiotic**	NT	WP_015704802.1	*eptA*	Phosphoethanolamine transferase	547	100	100	KEGG, Prokka, BLAST
		WP_047038023.1	*eptB*	Phosphoethanolamine transferase	563	91.16	98.08	KEGG, Prokka, BLAST
		WP_020077750.1	*arnT*	Phosphoethanolamine transferase	551	89.47	100	KEGG, Prokka, BLAST

NT (not tested), susceptibility testing was not performed. The DNA fragments verified by sequencing corresponded to the genes identified in whole genome sequencing.

**Figure 5:**
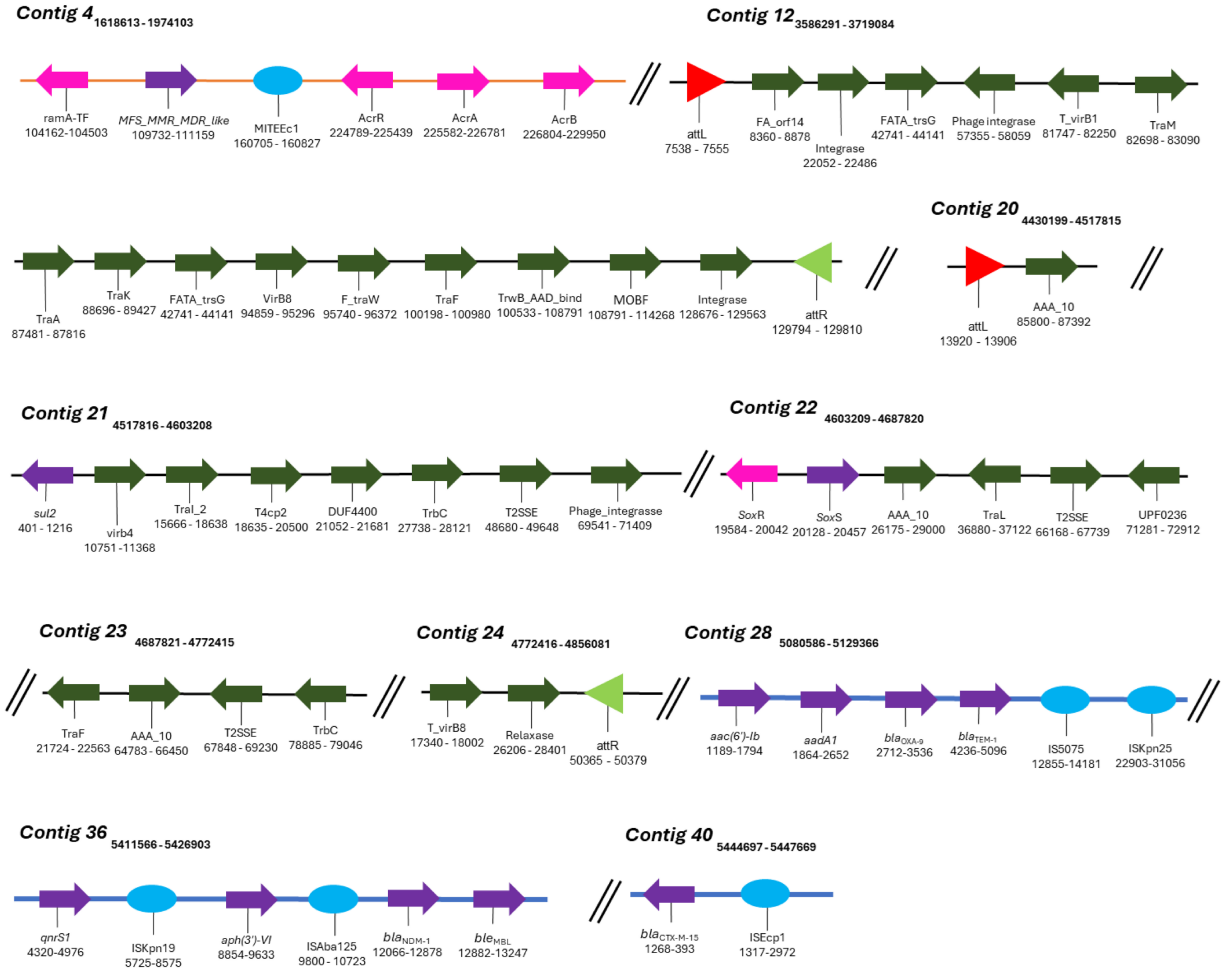
Mobile genetic elements (MGEs) identified in *K. aerogenes* CRKA317. Schematic representation of MGEs identified. The orange line represents the DNA strand (contig 4), blue lines represent GIs and black lines (contig 12 and contigs 20 to 24) represent the ICEs. The colored arrows represent the following genes: dark green - conjugation of the ICEs (e.g., contig 12), purple – resistance genes (e.g., contig 28), pink – efflux pumps and their respective activators/regulators (e.g., contig 4). The red (contig 20) and light green triangles (contig 24) represent *attL* and *attR* sequences, respectively. The light blue circles represent insertion sequences (e.g., contig 40).

A rich repertoire of genes related to efflux-mediated resistance was found in the genome of *K. aerogenes* CRKA317 (Table 3), including an ATP-binding cassette (ABC) antibiotic efflux pumps (*tolC*), resistance-nodulation-cell division (RND)-type efflux pumps (*oqxA*, *oqxB*, *acrA*, *acrB, acrD, HAE1*, *EefA, EefB*, *mdtA, mdtB*), major facilitator superfamily membrane transport proteins (*mdtH, KdeA*, *MFS-MMR-*like), a multidrug and toxic compound extrusion transporter (MATE) (*mdtK*) and an outer membrane efflux protein (*oprM*). In addition, we have identified multiple MDR efflux pump acrAB transcriptional activators/regulators (*marR*, *ramA*, *soxS*), as well as genes that code for porin-associated proteins such as *oprD*, *ompA*, *ompX*, and *ompW*. The *ramA* and *soxS* genes were specifically associated with mobile genetic elements (Figure 5).

**Table 3:** Identification of the genes encoding multidrug efflux, activators/regulators and outer membrane proteins genes in the genome of *K. aerogenes* CRKA317.

Type	Antibiotic resistance	Reference sequence (NCBI)	Putative resistance genes	Resistance gene/protein, mechanism function	Size (aa)	Coverage (%)	aa identity (%)	Resistance gene characterization
**Multidrug Efflux**	Quinolones, Tigecycline	WP_015367128.1	*oqxA*	Multidrug efflux RND transporter periplasmic adaptor subunit A	391	100	84.35	CARD, ABRicate, ResFinder, Arg-Annot, KEGG, Prokka
		WP_047041493.1	*oqxB*	Multidrug efflux RND transporter periplasmic adaptor subunit B	1,050	99.30	88.25	CARD, ABRicate, ResFinder, Arg-Annot, KEGG, Prokka
	Tetracycline, Glycylcycline, Penam, Cephalosporin, Phenicol, Rifamycin, Fluoroquinolone, Tigecycline, Disinfecting Agents and Antiseptics	WP_047038885.1	*acrA*	Multidrug efflux pump subunit A	399	100	100	KEGG, Prokka, BLAST
		WP_015367916.1	*acrB*	Multidrug efflux pump subunit B	1,048	100	100	KEGG, Prokka, BLAST
		WP_032712139.1	*acrD*	Multidrug efflux pump subunit D	1,037	100	100	KEGG, Prokka, BLAST
	Not totally known	WP_047038579.1	*HAE1* Family Pump	Multidrug efflux RND transporter permease subunit	1,035	100	100	BLAST
	Chloramphenicol, Norfloxacin, Acriflavine	WP_015367543.1	*kdeA*	MdfA family multidrug efflux MFS transporter	410	100	100	BLAST
	Fluoroquinolone	WP_015367204.1	*mdtH*	Multidrug efflux MFS transporter	402	100	100	KEGG, Prokka, BLAST
	Aminocoumarin	WP_045367110.1	*mdtA*	MuxA family multidrug efflux RND transporter periplasmic adaptor subunit	414	100	100	KEGG, Prokka, BLAST
	Aminocoumarin	WP_270843647.1	*mdtB*	MuxB family multidrug efflux RND transporter periplasmic adaptor subunit	1,040	100	99.90	KEGG, Prokka, BLAST
	Fluoroquinolone	WP_015366890.1	*mdtK*	MdtK family multidrug efflux MATE transporter	457	100	100	KEGG, Prokka, BLAST
	Aminoglycosides, erythromycin	WP_063402362.1	*oprM*	Outer membrane efflux protein OprM	459	100	100	KEGG, Prokka, BLAST
	Methylenomycin	WP_047038952.1	*MFS-MMR-MDR-like*	Methylenomycin A resistance protein	475	100	100	Prokka, BLAST
	Chloramphenicol, Ciprofloxacin, Erythromycin, Tetracyclines	EIX9084829.1	*eefA*	Multidrug efflux RND transporter permease subunit A	374	99	99.73	BLAST
		WP_285197781.1	*eefB*	Multidrug efflux RND transporter permease subunit B	1,035	99	99.71	BLAST
**Multidrug efflux activators and regulators**	Tetracycline, Cephalosporin, Phenicol, Glycylcycline, Penam, Fluoroquinolone, Rifamycin, Monobactam, Cephamycin, Carbapenem	WP_015368734.1	*soxS*	Superoxide response transcriptional regulator	109	91	100	CARD, KEGG, Prokka, BLAST
	Cyprofloxacin, Tetracycline	WP_015366732.1	marR*	Multiple antibiotic resistance transcriptional regulator	144	100	100	CARD, KEGG, Prokka, BLAST
**Outer membrane proteins**	Carbapenems	VAG14479.1	*oprD*	Outer membrane porin D	450	100	99.33	Prokka, BLAST
	Cephalosporin, Carbapenem, Penam, Monobactam, Cephamycin	WP_042894578.1	*ompC* (3 copies)	Outer membrane protein OprC	380	100	100	BLAST
	Peptide antibiotic/ β-lactam	WP_270843755.1	*ompA_C-like*	Peptidoglycan binding domains similar to the C-terminal domain of outer-membrane protein OmpA	560	100	100	BLAST
		WP_080473199.1	*ompA*	Outer membrane protein A	350	100	100	Prokka, BLAST
		WP_015367577.1	*ompX*	Outer membrane protein X	171	100	100	Prokka, BLAST
		WP_015705753.1	*ompW*	Outer membrane protein W	212	100	100	Prokka, BLAST

^*^Mutations found in genes may indicate the presence of antibiotic resistance.

*Klebsiella aerogenes* CRKA317 presented amino acid substitutions in marR (Ser3Asn), which may play a role in the development of quinolone resistance (Maneewannakul and Levy, 1996). Additionally, we found more two novel mutations in marR (Val96Ile and Gly103Glu), and one novel mutation (Ala12Glu) in the transcription factor of the regulon, soxS (Supplementary Figure S2).

### Genomic islands, mobile genetic elements, and prophage

We searched the *K. aerogenes* CRKA317 genome for the presence of GIs and Mobile Genetic Elements (MGEs). GIs are groups of genes within a bacterial genome that appear to have been obtained through horizontal gene transfer. We found 22 GIs in *K. aerogenes* CRKA317’s genome, which contained both resistance genes and insertion sequences. It is noteworthy that these antimicrobial resistance genes (*bla*_OXA-9_, *bla*_TEM-1_, *bla*_NDM-1_, *ble*_MBL_, *bla*_CTX-M-15_, *aac(6’)-Ib*, *aadA/ant(3”)-Ia*, *aph(3’)-VI*, and *qnrS1*) were located within an island in the contigs of the *K. aerogenes* CRKA317 genome that did not align with the reference strain *K. aerogenes* 57 isolate (Supplementary Figure S3). Furthermore, only one resistance gene (*bla*_AmpC_) was identified in islands located within the aligned contigs with reference strain *K. aerogenes* 57.

MGEs consist of a broad array of genomic sequences, such as plasmids, prophages, pathogenicity islands, restriction and modification systems, transposons, and Insertion Sequences (ISs). Our analyses showed that the *K. aerogenes* CRKA317 genome contained eight families of ISs, two copies of putative integrative and conjugative elements (ICE) with type IV secretion system (T4SS), and eight prophage regions. ISs are mobile repetitive DNA sequences that have the capacity to replicate and relocate within a host genome, playing a role in genetic diversity and regulation of gene expression in prokaryotes (Tempel et al., 2022). Our CRK317 strain contained two copies of MITEEc1 from the IS630 family, albeit only one copy carried genes such as *MFS_MMR_MDR-like*, *acrR*, *acrA*, and *acrB* (contig 4). The IS110 family presented one copy of IS4321/IS5075 and one copy of ISKpn25 harboring the *aac(6’)-Ib*, *aadA1*, *bla*_OXA-9_, and *bla*_TEM-1_ genes (contig 28). Contig 36 comprised the IS30 family harboring one copy of ISAba125, carrying *bla*_NDM-1_ and *ble*_MBL_ genes and the ISKra4 family presenting one copy of ISKpn19, *aph(3’)-VI* and *qnrS1* genes. The ISEcp1 member of the IS1380 family presented the *bla*_CTX-M-15_ gene (contig 40) (Figure 5). The ISRaq1 member of the IS3 family, the ISR1 member of the IS1 family and the IS26 member of the IS6 family did not present any resistance genes.

ICEs are found in the bacterial host chromosome and play a significant role in spreading resistance genes which significantly contribute to the evolution of discrete bacterial strains (Bean et al., 2022). Among the two putative ICEs containing a type IV secretion system (T4SS), contig 12 exhibited elements features of the ICE backbone, including a 16-base pair direct repeat *attL* sequence (5’-AAGAAGGGGAGTCCTG-3’); various integrases such as virB(s), rve, and phage integrases; T4SSs; T4CP; relaxases; and another 16-base pair direct repeat *attrR* sequence (5’-AAGAAGGGGAGTCCTG-3’). Additionally, contigs 20-24 contained similar components with a slight variation in the length of its direct repeats, 15 base pairs for both *attL* and *attrR* (Table 4). We did not detect CRISPR or *Cas* genes in *K. aerogenes* CRKA317 genome.

**Table 4:** The genetic compositions of the MGEs predicted in *K. aerogenes* CKA317.

MGE type	Family	Contig	Position in contig	ID	Accession number	Program	Near resistance genes
**Insertion sequences** **(ISs)**	IS630	4	160705–160827	MITEEc1	U00096	TnCentral ISFinder	*ramA* *MFS_MMR_MDR-*like *acrR* *acrA* *acrB*
		16	75513–75586	MITEEc1	U00096	TnCentral ISFinder	–
	IS3	10	28955–30017	ISRaq1	AY528232	ISFinder	*–*
	IS110	28	12855–14181	IS4321/IS5075	AF457211	TnCentral MGEFinder	*aac(6’)-Ib* *aadA1* *bla*_OXA-9_ *bla*_TEM-1_
			22903–31056	ISKpn25	NC_009650	TnCentral MGEFinder	
	IS1	31	2536–3303	IS1R	J01730	TnCentral ISFinder	–
	ISKra4	36	5725–8575	ISKpn19	NC_010886	ISFinder MGEFinder	*qnrS1* *aph(3’)-VI*
	IS30	36	9800–10723	ISAba125	AY751533	TnCentral	*bla*_NDM-1_ *ble*_MBL_
	IS1380	40	1317–2972	ISEcp1	AJ242809	TnCentral ISFinder MGEFinder	*bla*_CTX-M-15_
	IS6	48	1–820	IS26	X00011	TnCentral ISFinder MGEFinder	–
**Integrative and conjugative elements (ICEs)**	T4SS	12	*attL*: 3593829–3593844 *attR*: 3716086–3716101	Putative ICE with T4SS	–	ICEFinder	–
		20–24	*attL*: 4500895–4500909 *attR*: 4822781–4822795	Putative ICE with T4SS	–	ICEFinder	*sul-2* *sox*S

Among the eight prophage regions identified in CRK317, two were completely intact, four were incomplete, and two were classified as uncertain. Similarly, two other phages were observed in the fully intact regions: the first region (43.2 kb in size with 50.3% CG content and 63 CDS) showed similarities to *Salmonella* phage SEN34 (NC_028699.1), while the second region (54.5 kb in size with 52.32% CG content and 54 CDS) exhibited similarities to *Escherichia* phage vB_EcoM_ECO1230-10 (NC_027995.1) (Supplementary Figure S4).

## Discussion

*Klebsiella aerogenes* is known as an opportunistic pathogen of patients admitted to the intensive care unit and is frequently associated with multidrug resistance (MDR) (Azevedo et al., 2018). It has been detected in various hospital sources and is known for its ability to adapt to this setting. This study found that *K. aerogenes* was mainly detected in rectal swabs and urine samples, followed by tracheal aspirate, blood, and catheter tips. Previous studies have highlighted that *K. aerogenes* is commonly present in human specimens such as urinary, gastrointestinal, respiratory, blood, abscesses, and cutaneous samples (Davin-Regli et al., 2019). Additionally, rectal swabs are employed for active surveillance of asymptomatic carriers. These findings are also consistent with prior studies demonstrating the presence of *E. aerogenes* in surveillance rectal swabs (Vrioni et al., 2012). But the small number of *K. aerogenes* isolates is one of the major limitations of this study.

Our study found that all *K. aerogenes* isolates displayed some level of resistance to the β-lactams tested, including carbapenems, and 50% of *K. aerogenes* isolates exhibited a MDR profile (Table 1). Carbapenems have traditionally been a preferred treatment for infections caused by MDR Gram-negative strains (Bouza, 2021). Therefore, the emergence of carbapenem-resistant *K. aerogenes* strains present a new challenge in the treatment of these infections and pose a public health threat worldwide (Kamio and Espinoza, 2022).

The presence of resistance genes was verified using polymerase chain reactions. Out of the 10 isolates, we discovered that two only contained the *bla*_KPC-2_ gene and three only contained the *bla*_NDM-1_ gene while, interestingly, five strains carried both the *bla*_KPC-2_ and *bla*_NDM-1_ genes. Previous studies have reported the presence of *bla*_KPC_ or *bla*_NDM_ genes in clinical *K. aerogenes* isolates from various countries (Pulcrano et al., 2016; Franolić et al., 2019; Ma et al., 2020), including Brazil (Bispo Beltrão et al., 2020; Soares et al., 2021). However, our literature review indicated that there has been only one study of *K. aerogenes* co-harboring both *bla*_KPC_ and *bla*_NDM_ genes, which was observed in China (Zhang et al., 2017). Therefore, to the best of our knowledge, this is the first report describing clinical samples of *K. aerogenes* isolated from Brazil with simultaneous carriage of *bla*_KPC_ and *bla*_NDM_ genes. It is noteworthy that we found a significant number of *K. aerogenes* isolates exhibiting a *bla*_KPC-2_ or *bla*_NDM-1_ carbapenemase in addition to ESBLs genes, including members of the *bla*_CTX-M-1_; *bla*_OXA1, 4, 30_; *bla*_TEM_; and *bla*_SHVvariants_. Our findings are in line with studies that reported the concomitant presence of carbapenemase and ESBLs genes in *K. aerogenes* strains (Ma et al., 2020; Pan et al., 2021).

Although most of the CRKA strains were susceptible to aminoglycosides (gentamicin and/or amikacin), a high percentage of the isolates were found to contain the *aph(3’)-VI* gene, a plasmid-encoded aminoglycoside phosphotransferase that confers resistance to amikacin. Studies have shown that *Enterobacter aerogenes* can carry both *aph(3’)-VI* and *bla*_KPC_ (Firmo et al., 2019), and also *aph(3’)-VI* with *bla*_NDM_ (Chen et al., 2015). However, to the best of our knowledge, this is the first report of *Klebsiella aerogenes* concomitant harboring *aph(3’)-VI*, *bla*_KPC-2_, and *bla*_NDM-1_ genes, as can be seen in the CRKA315 strain. Most of the CRKA isolates also harbored the *aac(6’)-Ib* gene, responsible for encoding an aminoglycoside 6’-N-acetyltransferase type Ib, which provides resistance to amikacin (Machuca et al., 2016). Interestingly, amikacin susceptible isolates harboring the *aac(6′)-Ib* gene have been reported in *K. pneumoniae* strains (Almaghrabi et al., 2014; Haldorsen et al., 2014; Galani et al., 2019). Despite half of the CRKA strains being susceptible to ciprofloxacin, 80% of the isolates harbored the *qnrS* (*qnrS1* and/or *S2*) gene. The *qnrS* genes have been detected in a variety of microorganisms and environments, where they can be located in both the chromosome and in plasmids (Xu et al., 2023). Studies have indicated an association between *qnrS1* and a Tn3-like-blaTEM-1-containing transposon, leading to enhanced recombination and insertion effectiveness (Guan et al., 2013). The expression of the *qnrS1* gene is enhanced by quinolones, in contrast to certain other *qnr* genes (Monárrez et al., 2018). *QnrS2*, associated with quinolone resistance which demonstrates a 92% similarity in amino acid composition with *qnrS1*, is frequently identified in IncQ, IncU, and ColE-type plasmids as part of a mobile insertion cassette element bracketing inverted repeats but lacking a transposase (Picão et al., 2008; Han et al., 2012; Dobiasova et al., 2016; Wen et al., 2016). Notably, a newly identified surrounding genetic structure of *qnrS2* flanked by IS26 elements was observed in *E. coli* strains from China (Tao et al., 2020). This finding highlights the important role of IS26 in facilitating the horizontal spread of quinolone resistance genes. Therefore, our data suggests that the variance in results between phenotyping and genotyping may be linked to the presence of multiple concurrent resistance mechanisms (Galani et al., 2019). However, we should mention that the incomplete sequencing of *K. aerogenes* CRKA317 may undermine the confidence level of this speculation.

Efflux pumps are important membrane proteins that play a crucial role as defense mechanisms by actively exporting harmful substances, such as antibiotics, detergents, and heavy metals (Jang, 2023). In our study, all of the isolates studied harbored *acrA*, encoding a subunit that functions as an adapter protein linked to AcrB, and TolC outer membrane channel proteins. Although we did not identify *acrB* in our analysis, together these proteins form the AcrAB-TolC stable efflux complex, known to contribute to multidrug resistance in nosocomial pathogens (Chen et al., 2022). These genes have been identified in *K. aerogenes* clinical isolates and are responsible for expelling various compounds, including antimicrobial agents like quinolones, tetracyclines, and chloramphenicol (Pradel and Pagès, 2002; Masi et al., 2007; Chevalier et al., 2008). Although the expression levels of *acrAB* and *tolC* genes were not determined in our *K. aerogenes* isolates, it was observed that all strains carried both genes which might be involved in the development of MDR carbapenem-resistant profile of *K. aerogenes*. The *mdtK* gene encodes an efflux pump that has the ability to expel acriflavine, doxorubicin, norfloxacin, and dipeptides (Hayashi et al., 2010; Andersen et al., 2015). This gene was found in a majority of our CRKA strains. Previous studies have reported the presence of the *mdtK* gene in *K. aerogenes* isolated from river sediment (Iyer et al., 2017).

WGS is a valuable tool for identifying and characterizing disease-associated bacteria in clinical settings. Thus, we conducted a comprehensive analysis of the entire genome of *K. aerogenes* CRKA317 to gain deeper insights into its genomic diversity, and methods of resistance.

The draft genome of *K. aerogenes* CRKA317 comprised of a single circular chromosome with a length similar to most *K. aerogenes* genomes in NCBI GenBank and harbored various essential genes for bacterial cellular processes. Additionally, the results of the RAST and eggNOG analyses showed that our *K. aerogenes* CRKA317 carried genes linked to drug resistance.

Next, we explored the phylogenetic affiliation of *K. aerogenes* CRKA317. The16S rRNA gene sequence analysis showed that the *K. aerogenes* CRKA317 was not closely related to *K. aerogenes* as a specie. Although, 16S rRNA gene is used extensively in bacterial phylogenetics, the limitations of using 16S rRNA gene relatedness to classify bacteria have been extensively documented (Rossi-Tamisier et al., 2015; Thorell et al., 2019; Soares et al., 2023). Therefore, a combination of ANI, dDDH, and TYGS technologies were employed to determine the phylogenetic position of the *K. aerogenes* CRKA317, which predicted a close phylogenetic relationship with *K. aerogenes*.

In our investigation of the genetic relationship between *K. aerogenes* CRKA317 and another 26 *K. aerogenes* strains, we found three strains (*K. aerogenes* 57, *K. aerogenes* CAVI1320, and *K. aerogenes* EA46506) that were closely related to *K. aerogenes* CRKA317. When analyzing the distribution of shared gene families among four strains, we found that our *K. aerogenes* CRKA317 had the highest number of singleton genes compared to its closest three relatives. Some of these singleton genes were related to antibiotic resistance. Singleton genes are typically acquired through horizontal gene transfer (HGT) or mutations in pre-existing genes. These genes are often associated with specific metabolic pathways, virulence, antibiotic resistance mechanisms, or other environmental adaptations (Costa et al., 2020). Furthermore, the KEGG pathway analysis revealed that most of the genes were in the core genome and were related to metabolic pathways. As the analyses were based on small number of *K. aerogenes*, we cautiously speculated that *K. aerogenes* might have genomic plasticity, that may contribute to antibiotic resistance and environmental adaptation.

Our comprehensive analysis of the *K. aerogenes* CRKA317 using WGS confirmed the correlation between the genotype and the phenotype to its antimicrobial resistance. Furthermore, our analysis found a large number of antibiotic resistance-associated genes such as porin and efflux pump-encoding genes giving resistance to both previously tested and untested antibiotics, suggesting a wide-ranging antibiotic resistance profile (Tables 2, 3). AmpC β-lactamases are usually encoded within the chromosome or found as *ampC* genes on a plasmid. Our *K. aerogenes* CRKA317 harbored two copies of *bla*_AmpC_ gene. Several Gram-negative organisms, including *E. aerogenes*, *Enterobacter cloacae*, *Serratia marcescens*, *Providencia stuartii*, *Pseudomonas aeruginosa*, *Hafnia alvei*, and *Morganella morganii*, have presented *AmpC* in their genomes (Jacoby, 2009; Ghanavati et al., 2018; Tamma et al., 2019). This enzyme provides resistance against aminopenicillins, cephalosporins, oxyimino-cephalosporins (e.g., ceftriaxone, cefotaxime, and ceftazidime), cephamycins (e.g., cefoxitin and cefotetan), and monobactams (aztreonam) (Jacoby, 2009). The gene *sul-2* implicated in sulphonamide resistance due to inducing high levels of dihydropteroate synthase was found in our strain (Teichmann et al., 2014). This gene has been widely studied and its association with sulfamethoxazole resistance has been demonstrated in numerous studies worldwide (Teichmann et al., 2014; Shin et al., 2015), including in an *E. aerogenes* from Brazil (Grazziotin et al., 2016). The *K. aerogenes* CRKA317 contained the *fosA* gene, which is commonly found in the genomes of *K. pneumoniae*, *K. oxytoca*, *E. cloacae*, *E. aerogenes*, *S. marcescens*, *M. morganii*, *P. stuartii*, and *P. aeruginosa*. All these species haboring *fosA* gene presented intrinsic resistance or reduced susceptibility to fosfomycin (Ito et al., 2017). The *ermC* gene, known for its role in conferring erythromycin resistance in *S. aureus* and other *Staphylococci* (Jamrozy et al., 2017) was also detected in the *K. aerogenes* CRKA317. The *K. aerogenes* CRKA317 also contained the *catA-like* gene, responsible for producing a chloramphenicol acetyltransferase that catalyzes chloramphenicol (Huang et al., 2017). This gene is commonly present on transposons and plasmids, and it is widespread among a range of organisms such as *Acinetobacter* spp., *Bacillus methylotrophicus* and *Chryseobacterium indologenes* (Obayiuwana and Ibekwe, 2020; Damas et al., 2022).

We also detected three alterations in amino acids in marA in our *K. aerogenes* CRKA317 (Ser3Asn, Val96Ile, Gly103Glu) and one in soxS (Ala2Glu). These findings partially corroborate the results of Maneewannakul and Levy (1996), where they identified three mutations in marA (Ser3Asn, Val96Glu, Gly103Ser) leading to resistance to fluoroquinolones in *E. coli*. Moreover, Aly et al. (2015) demonstrated that a mutation in soxS (Ala12Ser) contributed to resistance against ciprofloxacin, enrofloxacin, chloramphenicol, and doxycycline in *E. coli* strains. We suggest these same mechanisms gave rise to antimicrobial resistance in our *K. aerogenes* CRKA317 due to the similar mutations noted.

Additionally, our *K. aerogenes* CRKA317 presented several MGEs harboring resistance genes (Table 4). MGEs are important tools for acquiring resistance genes through horizontal gene transfer. ISs, for example, are transposable DNA segments that have been previously linked to resistance genes and can be transferred horizontally by plasmids or by bacteriophages (Siguier et al., 2014). The *bla*_OXA-9_, *bla*_TEM-1_ genes encoding for resistance to β-lactams, and *aac(6’)-Ib*, *aadA1* encoding resistance aminoglycosides were related to an IS110 family transposase (IS4321/IS5075 and ISKpn25 insertion elements). These findings are partially supported by previous studies that have shown ESBL-encoding genes (*bla*_TEM-1B_) and genes related to aminoglycoside resistance (*aph-Id*, *aph-Ib*) are located near to an IS5075 insert in *K. pneumoniae* (Pajand et al., 2023). The *bla*_CTX-M-15_ gene was in close proximity to ISEcp1, which is a member of the IS1380 family (Poirel et al., 2008), and has been identified as one of several elements responsible for facilitating the transfer of *bla*_CTX-M_ genes across various species of *Enterobacteriaceae* (Shawa et al., 2021; Wang et al., 2023). The *bla*_NDM-1_ and *ble*_MBL_ genes (a class B3 β-lactamases with carbapenemase activity) were close to the insertion sequence *ISA*ba125 (*IS*30 family). Studies have proposed that the *ISA*ba125–*ble*_NDM_ combination occurred initially in *Acinetobacter* spp. and later transferred to other Gram-negative bacteria (Castanheira et al., 2023). Additionally, the *ISA*ba125–*bla*_NDM-1_–*ble*_MBL_ combination has been found in a structure referred to as NDM-GE-U.S, first observed in a *K. pneumoniae* strain from the United States and subsequently detected in various strains worldwide (Hudson et al., 2014; Peirano et al., 2018).

The *qnrS1* the *aph(3”)-VI* genes, were found in close proximity to the I*SK*pn19 insertion sequence of the *ISK*ra4 family in our isolate. Studies have shown that *qnrS1* gene, which confers resistance to ciprofloxacin, is related to *ISK*pn19 in various bacteria, such as *Leclercia adecarboxylata*, *Salmonella corvallis; K. pneumoniae*, *E. coli*, and *S. marcescens* (Xu et al., 2020; Chen et al., 2023; Sano et al., 2023; Zheng et al., 2024). The *aph(3”)-VI* gene, responsible for amikacin modification, was not found to be flanked by the I*SK*pn19 insertion sequence in our literature review. It is worth noting that *K. aerogenes* CRKA317 presents a large number of singleton genes, including *bla*_NDM-1_, *aadA/ant(3”)-Ia*, *aph(3’)-VI*, and *qnrS1* and its presence indicates the variability and diversity within the genome of our strain. It is possible that these genes were obtained from other lineages through horizontal gene transfer, leading to the development of new functions, such as resistance to antibiotics. This assumption can be reinforced by the presence of a genomic island that carries most of the resistance genes (*bla*_OXA-9_, *bla*_TEM-1_, *bla*_NDM-1_, *ble*_MBL_, *bla*_CTX-M-15_, *aac(6’)-Ib*, *aadA/ant(3”)-Ia*, *aph(3’)-VI*, and *qnrS1*) found in our genome. Genomic islands are distinct regions in the bacterial genome with genes related to each other and often associated with specific functions. These islands are often acquired through horizontal gene transfer events and are associated with the widespread distribution of antimicrobial resistance factors among bacteria (Juhas et al., 2009). The composition of genomic islands is conducive to the acquisition of new antibiotic resistance genes, as they include several MGEs, which facilitate the incorporation of new genes, but also their own transfer, for example, using tRNA genes as recombination sites into the chromosome (da Silva Filho et al., 2018).

Our *K. aerogenes* CRKA317 displayed many genes of interested linked to antibiotic resistance (Table 3). The *ramA*, *acrR*, *acrA* and *acrB* genes, and *MFS_MMR_MDR-like* gene were found to be associated with MITEEc. MITEEc belongs to the IS630 family and was found in an extensively drug-resistant (XDR) *Escherichia coli* isolate (Jain et al., 2021). *RamA* belongs to the AraC/XylS protein family and shows a close association with the marA and soxS proteins (Rosenblum et al., 2011). Elevated expression of *ramA* is associated with the activation of the acrAB efflux pump, which confers multidrug resistance in various bacterial species such as *E. aerogenes*, *K. pneumoniae*, and *E. cloacae* (Chollet et al., 2004; Keeney et al., 2007; Ruzin et al., 2008). The *acrR* gene regulates the multidrug efflux pump AcrAB-TolC (Subhadra et al., 2018). The *MFS_MMR_MDR-like* gene is linked to methylenomycin resistance, and the antibiotic Methylenomycin A is produced naturally by *Streptomyces coelicolor* A3, a model organism for streptomycetes (Bentley et al., 2002; Bowyer et al., 2017). The *acrAB-tolC* system where the acrAB fusion protein, members of the RND-type efflux family, function with another antibiotic efflux pump, *tolC*, to pump out various compounds such as SDS, novobiocin, deoxycholate, aminoglycosides, and dianionic β-lactams including carbenicillin, oxacillin, nafcillin, and aztreonam (Guérin et al., 2023). We also found many other RND antibiotic efflux pumps, the first pair being *eefA* and *eefB* which are part of the *eefABC* locus known to encode a tripartite efflux pump that gives rise to resistance to erythromycin and other antibiotics in *E. aerogenes* (Pradel and Pagès, 2002; Masi et al., 2007). The *oqxA* and *oqxB* operon has also been described to increase antibiotic resistance in *E. aerogenes*, this time to quinolones as they combine to make the oqxAB efflux pump (Wong et al., 2015; Moosavian et al., 2021). The HAE1 family, also contained in our isolate, contains large number of identified RND transporters (Nikaido, 2018). These pumps are commonly found in Gram-negative bacteria, typically existing as trimers, and are involved in the transportation of drugs and other hydrophobic substances (Nikaido, 2018). In addition, we found members of the acrB/acrD/acrF family, specifically *acrD*, *mdtA* and *mdtB*. The *mdt*ABC operon is transcriptionally activated by *baeR* and leads to the formation of the mdtABC tripartite complex, which provides resistance to novobiocin and deoxycholate in *E. coli* (Nagakubo et al., 2002). Related to this complex, it was found *mdtH* and *mdtK* both of which give rise to proteins that function has multidrug efflux pumps that contribute to resistance against quinolone antibiotics such as norfloxacin and enoxacin in *E. coli* strains (Nishino and Yamaguchi, 2001; Yu et al., 2020). The final RND antibiotic efflux pump sequenced in our isolate is *oprM* which is part of MexX-MexY-OprM efflux systems that mediate intrinsic antibiotic resistance to aminoglycosides and erythromycin in bacteria such as *P. aeruginosa* (Wong et al., 2001), *Brevundimonas brasiliensis* sp. nov and *Burkholderia vietnamiensis* (Shinoy et al., 2013; Soares et al., 2023).

We also found putative ICE with T4SS just harboring *sul-2* gene, encoding for resistance to sulfonamide, and *sox* gene, a key component of a central regulatory system present in all *Enterobacteriaceae*, which detects and reacts to internal chemical stressors like antibiotics (Chubiz, 2023). ICEs are mobile elements integrated into the chromosomes that can be excised and transferred horizontally to other bacteria and, therefore, have been associated with antimicrobial resistance genes (Johnson and Grossman, 2015). Despite this, due to the finding of only one resistance gene in the ICEs studied here (*sul-2*), we can assume that this structure is not the main source of resistance gene acquisition in *K. aerogenes* CRKA317.

Genes associated with porins were the second category identified in our isolate (Table 3). Porins belong to a category of transmembrane proteins called omps, which form small channels in the membrane and facilitate the passive movement of hydrophilic compounds. They regulate cellular permeability and can either enhance or reduce resistance to antibiotics. In our strain, we specifically found the outer membrane protein encoding genes *oprD, ompC, ompA, ompX* and *ompW* which have been found to have clinical significance. For instance, a reduction or absence of OmpC in clinical *E. aerogenes* isolates has been linked to a slight increase in imipenem MIC (Lavigne et al., 2012). Meanwhile, overexpressing ompX in *E. aerogenes* results in elevated resistance to β-lactam antibiotics, possibly due to significant reduction in the Omp36 porin (Hejair et al., 2017). *Omp*W expression in *A. baumannii* isolates was found to increase when exposed to ciprofloxacin and decrease when exposed to imipenem (Gurpinar et al., 2022). Conversely, *A. baumannii* strains with mutations in ompA exhibited reduced permeability for cephalothin/cephaloridine and lower minimum inhibitory concentrations for a range of antibiotics including imipenem, colistin, meropenem, chloramphenicol, aztreonam, and nalidixic acid (Smani et al., 2014; Tsai et al., 2020). Finally, in *P. aeruginosa*, the porin oprD plays a significant role in the uptake of basic amino acids and carbapenems (Wong et al., 2001).

Other genes of interest included: a superoxide response transcriptional regulator (*soxS*), a multiple antibiotic resistance transcriptional regulator (*marA*), and a major facilitator superfamily member (*kdeA*),

Prophages play a role in the survival mechanisms of their hosts and contribute to the enhancement of genetic diversity within the host genome (Kondo et al., 2021). In our study, we found two intact regions which were associated with the presence of a prophage highly similar to the *Salmonella* phage SEN34 (National Center for Biotechnology Information reference sequence NC_028699.1), and *Escherichia* phage vB_EcoM_ECO1230-10 (NC_027995.1). Prophage regions of *Salmonella* phage SEN34 (NC_028699.1) has been identified in *Salmonella salamae* (Hounmanou et al., 2022), and *Salmonella enterica* serovar Paratyphi B (Castellanos et al., 2020) and have been linked to drug resistance.

There are limitations to our study that need to be acknowledged. We encountered difficulties in assembling complete plasmid sequences, primarily due to the short reads generated by high-throughput sequencer. This can result in antimicrobial resistance genes being located on incomplete contigs, leading to uncertainty about whether they are situated on a plasmid or within the chromosome (Orlek et al., 2017; Berbers et al., 2020). Nonetheless, it is important to highlight those studies conducted in China have shown that clinical isolates of carbapenem-resistant *K. aerogenes* carried *bla*_NDM-1_ gene on plasmids of the IncFIIAs type. In another study, Shen et al. (2019) identified a plasmid (p1564) containing genes for plasmid replication (*Inc*A/C *rep*A), antibiotic resistance (*bla*_NDM-1_, *rmtC*, *aacA4*, *ble*_MBL_, *bla*_CMY-6_ and *sul-1*), and conjugation (*tra* clusters). In *K. aerogenes*, there is still no consensus on the location of the *bla*_KPC-2_ gene in the plasmid, the transposon variants capable of carrying this gene, and which incompatibility (Inc) groups carry the *bla*_KPC_ gene (Bispo Beltrão et al., 2020). However, a study conducted by Bispo Beltrão et al. (2020) in Brazil has described a non-Tn4401 element (NTEKPC-IId) that carries the *bla*_KPC-2_ and *aph(3’)-VII* genes in *Inc*Q1 plasmids in *K. aerogenes*. To date, the *Inc*Q1 *bla*_KPC-2_-positive plasmids have been found in different strains such as *E. coli*, *K. pneumoniae* of CG258, *Klebsiella quasipneumoniae*, and *P. aeruginosa* (de Oliveira Santos et al., 2018).

Although *K. aerogens* has not been reported to carry the plasmids found in our sequencing study, it is important to note genes such as *bla*_KPC_ and *bla*_NDM_ are commonly associated with the plasmid fragments found in *K. aerogenes* CRKA317. Takei et al. (2022) found nine isolates of *K. pneumoniae* carrying *bla*_NDM-1_ and *bla*_CTX-M-15_ on the IncFIB (pQil) plasmid and another five isolates carrying *bla*_NDM-1_ on the IncC plasmid. Similar data were also observed in the results of Zeng et al. (2022), who identified the *bla*_NDM-1_ gene in IncC plasmids from 21 *K. pneumoniae* isolates. Finally, the fragmented INCFIIK plasmid observed in our genome has already been noted to carry genes such as *bla*_KPC-2_, *bla*_CTX-M-15_, *bla*_TEM-1_ and, less commonly, *bla*_NDM-1_ (Chen et al., 2013; Bi et al., 2018). This suggests an emerging mechanism, using Inc. groups, that plays a role in the dissemination of carbapenem resistance in clinically important bacteria.

In conclusion, our current research has uncovered a concerning scenario involving *K. aerogenes* demonstrating resistance to commonly utilized drugs for treating infections, including those considered as last-resort options for life-threatening infections in ICU patients. Moreover, the presence of mobile genetic elements highlights the alarming potential for the transmission of various resistance genes such as *bla*_NDM-1_ and *bla*_KPC-2_ within hospital settings to susceptible populations. This scenario poses significant challenges for managing infectious diseases and underscores the necessity of early detection of such genetic features or mutations.

Our study did not involve human genetic material or biological samples. The strains were obtained from the collection of the Central Laboratory of Public Health, a leading diagnostic center in Tocantins, Brazil. This was a retrospective study and epidemiological data were obtained from a database at LACEN-TO in accordance with Resolution 466/12 of the National Health Council (Conselho Nacional de Saúde/Ministério da Saúde, 2012). Informed consent was not required as per Resolution 466/12 regarding research involving humans by the National Health Council. The study received approval from the Committee of Ethics in Human Research at the Federal University of São Carlos (no. 1.088.936), and permissions to conduct it were obtained from the State Department of Health in Tocantins and LACEN/TO.

## Data availability statement

The datasets presented in this study can be found in online repositories. The names of the repository/repositories and accession number(s) can be found in the article/Supplementary material.

## Ethics statement

The studies involving humans were approved by Committee of Ethics in Human Research at the Federal University of São Carlos (no. 1.088.936). The studies were conducted in accordance with the local legislation and institutional requirements. Written informed consent for participation was not required from the participants or the participants’ legal guardians/next of kin in accordance with the national legislation and institutional requirements.

## Author contributions

SR: Formal analysis, Investigation, Methodology, Writing – review & editing. GN: Formal analysis, Investigation, Methodology, Writing – review & editing. GS: Formal analysis, Investigation, Methodology, Writing – review & editing. MD: Formal analysis, Investigation, Methodology, Writing – review & editing. RF: Formal analysis, Investigation, Methodology, Writing – review & editing. PL: Formal analysis, Investigation, Methodology, Writing – review & editing. RS: Formal analysis, Methodology, Writing – review & editing. LC: Methodology, Writing – review & editing. AC: Formal analysis, Writing – review & editing. IM: Visualization, Writing – review & editing. AC: Visualization, Writing – review & editing. M-CP: Conceptualization, Funding acquisition, Project administration, Supervision, Writing – original draft, Writing – review & editing.

## Footnotes

^1^ https://www.bioinformatics.babraham.ac.uk/projects/fastqc/

^2^ https://www.bioinformatics.babraham.ac.uk/projects/trim_galore/

^3^ https://cge.food.dtu.dk/services/PlasmidFinder/

^4^ https://proksee.ca/

^5^ https://rast.nmpdr.org/

^6^ http://eggnog-mapper.embl.de/

^7^ https://www.kegg.jp/blastkoala/

^8^ https://www.ebi.ac.uk/Tools/msa/mafft/

^9^ https://ggdc.dsmz.de/ggdc.php

^10^ https://discover.nci.nih.gov/cimminer/oneMatrix.do

^11^ https://tygs.dsmz.de/

^12^ https://realphy.unibas.ch/realphy/

^13^ https://orthovenn2.bioinfotoolkits.net/task/create

^14^ https://card.mcmaster.ca/analyze/rgi

^15^ http://genepi.food.dtu.dk/resfinder

^16^ https://galaxy.pasteur.fr/

^17^ https://www.kegg.jp/blastkoala/

^18^ https://www.pathogenomics.sfu.ca/islandviewer/

^19^ https://tncentral.ncc.unesp.br/

^20^ https://www-is.biotoul.fr/index.php

^21^ https://cge.food.dtu.dk/services/MobileElementFinder/

^22^ https://bioinfo-mml.sjtu.edu.cn/ICEfinder/ICEfinder.html

^23^ https://crisprcas.i2bc.paris-saclay.fr/CrisprCasFinder/Index

^24^ https://phaster.ca/

^25^ https://www.ncbi.nlm.nih.gov/sra

^26^ https://www.ncbi.nlm.nih.gov/
