# Supplementary material for: First report of coexistence of blaKPC-2 and blaNDM-1 in carbapenem-resistant clinical isolates of Klebsiella aerogenes in Brazil
Source: Front Microbiol. 2024 Feb 14;15:1352851. doi: 10.3389/fmicb.2024.1352851 (PMC10903355; doi:10.3389/fmicb.2024.1352851)
Supplement: Supplementary file 1 [file Table_1.DOCX]

**SUPPLEMENTARY MATERIAL**

**Table S1.** Sequences of primers used for the identification of resistance genes.

| **Genes related to antibiotic resistance** | | | | | |
| --- | --- | --- | --- | --- | --- |
|  | **Gene** | **Sequence (5′-3′), F/R** | **TM (°C)** | **Amplicon size (bp)** | **Reference** |
| β-Lactam | *bla*_TEM_ | TGCGGTATTATCCCGTGTTG  TCGTCGTTTGGTATGGCTTC | 63 | 296 | (Xiong et al., 2007) |
|  | *bla*_CTX-M-1 group (CTX-M-1, -3,-10,-11 and -12)_ | ACAGCGATAACGTGGCGATG  TCACCCAATGCTTTACCCAG | 64 | 216 | (Xiong et al., 2004) |
|  | *bla*_SHV variants_ | AGCCGCTTGAGCAAATTAAAC  ATCCCGCAGATAAATCACCAC | 55.6 | 712 | (Dallenne et al., 2010) |
|  | *bla*_OXA-1, OXA-4 and OXA-30_ | GGCACCAGATTCAACTTTCAAG  GACCCCAAGTTTCCTGTAAGTG | 63 | 563 |  |
|  | *bla*_GES-1_ *to bla*_GES-9_ and *bla*_GES-11_ | AGTCGGCTAGACCGGAAAG  TTTGTCCGTGCTCAGGAT | 57 | 399 |  |
|  | *bla*_PER-1_ *and bla*_PER-3_ | GCTCCGATAATGAAAGCGT  TTCGGCTTGACTCGGCTGA | 60 | 520 |  |
|  | *bla*_VEB-1_ *to bla*_VEB-6_ | CATTTCCCGATGCAAAGCGT  CGAAGTTTCTTTGGACTCTG | 60 | 648 |  |
|  | *bla*_KPC_ | CGTCTAGTTCTGCTGTCTTG  CTTGTCATCCTTGTTAGGCG | 61.3 | 797 | (Poirel et al., 2008) |
|  | *bla*_OXA-48_ | GCGTGGTTAAGGATGAACAC  CATCAAGTTCAACCCAACCG | 55 | 438 |  |
|  | *bla*_IMP-1_ | CTACCGCAGCAGAGTCTTTGC  ACAACCAGTTTTGCCTTACC | 55 | 587 | (Martins et al., 2007) |
|  | *bla*_VIM-2_ | AAAGTTATGCCGCACTCACC  TGCAACTTCATGTTATGCCG | 55 | 865 | (Yan et al., 2001) |
|  | *bla*_NDM_ | GCAGCTTGTCGGCCATGCGGGC  GGTCGCGAAGCTGAGCACCGCAT | 60 | 782 | (Doyle et al., 2012) |
|  | *bla*_SPM-1_ | AAAATCTGGGTACGCAAACG  ACATTATCCGCTGGAACAGG | 52 | 271 | (Ellington et al., 2006) |
|  | *bla*_GIM-1_ | TCGACACACCTTGGTCTG AA  AACTTCCAACTTTGCCAT GC | 52 | 477 |  |
|  | *bla*_SIM-1_ | TACAAGGGATTCGGCATC  TAATGGCCTGTTCCCATGT | 52 | 570 |  |
| Aminoglycosides | *armA* | ATTCTGCCTATCCTAATTGG  ACCTATACTTTATCGTCGTC | 55 | 315 | (Sheikhalizadeh et al., 2017) |
|  | *rmtB* | ATGAACATCAACGATGCCCT  CCTTCTGATTGGCTTATCCA | 55 | 769 | (Yan et al., 2004) |
|  | *aph(3′)-VIa (aphA6)*  *aac-(6’)-Ib* | CGGAAACAGCGTTTTAGA  TTCCTTTTGTCAGGTC  TATGAGTGGCTAAATCGAT  CCCGCTTTCTCGTAGCA | 49  53 | 716  395 | (Noppe-Leclercq et al., 1999)  (Jinwei Huang, 2012) |
| Tetracycline | *tetB* | CAGTGCTGTTGTTGTCATTAA  GCTTGGAATACTGAGTGTAA | 59.7 | 571 | (Call et al., 2003) |
| Colistin | *mcr-1* | CGGTCAGTCCGTTTGTTC  CTTGGTCGGTCTGTAGGG | 51.6 | 309 | (Liu et al., 2016) |
| Sulfonamide | *sul-1* | CGGCGTGGGCTACCTGAACG  GCCGATCGCGTGAAGTTCCG | 69 | 433 | (Kerrn, 2002) |
|  | *sul-2* | GCGCTCAAGGCAGATGGCATT  GCGTTTGATACCGGCACCCGT | 69 | 293 |  |
| Plasmid-mediated quinolone resistance (PMQR) | *aac(6′)-Ib-cr* | ATGACTGAGCATGACCTTGC  TTAGGCATCACTGCGTGTTC | 55.4 | 519 | (Platell et al., 2011) |
|  | *qnrS (qnrS1 and 2)* | GCAAGTTCATTGAACAGGGT  TCTAAACCGTCGAGTTCGGCG | 54 | 428 | (Cattoir et al., 2007) |
| Multidrug efflux pump | *acrAB* | ATCAGCGGCCGGATTGGTAAA  CGGGTTCGGGAAAATAGCGCG | 53 | 312 | (Wasfi et al., 2016) |
|  | *tolC* | ATCAGCAACCCCGATCTGCGT  CCGGTGACTTGACGCAGTCCT | 51 | 520 | (Wasfi et al., 2016) |
|  | *mdtK* | GCGCTTAACTTCAGCTCA  GATGATAAATCCACACCAGAA | 43 | 453 | (Wasfi et al., 2016) |

*The letters F and R indicate the forward and reverse primer sequences, while TM denotes the melting temperature employed in PCR amplification.

**Table S2:** 16S rRNA sequences used to assemble the 16S rRNA phylogenetic tree

| Sample/Species | NCBI RefSeq |
| --- | --- |
| *Klebsiella quasivariicola* strain KPN1705 16S ribosomal RNA | NR_181901.1 |
| *Klebsiella pasteurii* strain SPARK836C1 16S ribosomal RNA | NR_180640.1 |
| *Klebsiella africana* strain SB5857 16S ribosomal RNA | NR_180233.1 |
| *Klebsiella huaxiensis* strain WCHKl090001 16S ribosomal RNA | NR_171417.1 |
| *Klebsiella grimontii* strain SB73 16S ribosomal RNA | NR_159317.1 |
| *Klebsiella quasipneumoniae* subsp. similipneumoniae strain 07A044 16S ribosomal RNA | NR_134063.1 |
| *Klebsiella quasipneumoniae* subsp. quasipneumoniae strain 01A030 16S ribosomal RNA | NR_134062.1 |
| *Klebsiella michiganensis* strain W14 16S ribosomal RNA | NR_118335.1 |
| *Klebsiella aerogenes* KCTC 2190 16S ribosomal RNA | NR_102493.2 |
| *Klebsiella pneumoniae* strain ATCC 13883 16S ribosomal RNA | NR_119278.1 |
| *Klebsiella oxytoca* strain ATCC 13182 16S ribosomal RNA | NR_119277.1 |
| *Klebsiella pneumoniae* subsp. ozaenae strain ATCC 11296 16S ribosomal RNA | NR_119276.1 |
| *Klebsiella oxytoca* strain ATCC 13182 16S ribosomal RNA | NR_118853.1 |
| *Klebsiella aerogenes* strain ATCC 13048 16S ribosomal RNA | NR_118556.1 |
| *Klebsiella pneumoniae* strain DSM 30104 16S ribosomal RNA | NR_117686.1 |
| *Klebsiella pneumoniae* strain DSM 30104 16S ribosomal RNA | NR_117685.1 |
| *Klebsiella pneumoniae* strain DSM 30104 16S ribosomal RNA | NR_117684.1 |
| *Klebsiella pneumoniae* strain DSM 30104 16S ribosomal RNA | NR_117683.1 |
| *Klebsiella pneumoniae* strain DSM 30104 16S ribosomal RNA | NR_117682.1 |
| *Klebsiella aerogenes* strain NCTC10006 16S ribosomal RNA | NR_114737.1 |
| *Klebsiella pneumoniae* strain DSM 30104 16S ribosomal RNA | NR_114715.1 |
| *Klebsiella pneumoniae* subsp. rhinoscleromatis ATCC 13884 16S ribosomal RNA | NR_114507.1 |
| *Klebsiella pneumoniae* strain ATCC 13883 16S ribosomal RNA | NR_114506.1 |
| *Klebsiella oxytoca* strain NBRC 102593 16S ribosomal RNA | NR_114152.1 |
| *Klebsiella pneumoniae* strain NBRC 14940 16S ribosomal RNA | NR_113702.1 |
| *Klebsiella aerogenes* strain NBRC 13534 16S ribosomal RNA | NR_113614.1 |
| *Klebsiella oxytoca* strain JCM 1665 16S ribosomal RNA | NR_113341.1 |
| *Klebsiella pneumoniae* strain JCM 1662 16S ribosomal RNA | NR_113240.1 |
| *Klebsiella oxytoca* strain JCM 1665 16S ribosomal RNA | NR_112010.1 |
| *Klebsiella pneumoniae* strain JCM1662 16S ribosomal RNA | NR_112009.1 |
| *Klebsiella pneumoniae* subsp. ozaenae strain ATCC 11296 16S ribosomal RNA | NR_041750.1 |
| *Klebsiella oxytoca* strain ATCC 13182 16S ribosomal RNA | NR_041749.1 |
| *Klebsiella pneumoniae* strain DSM 30104 16S ribosomal RNA | NR_036794.1 |
| *Klebsiella aerogenes* strain JCM 1235 16S ribosomal RNA | NR_024643.1 |
| *Klebsiella variicola* strain F2R9 16S ribosomal RNA | NR_025635.1 |
| *Klebsiella pneumoniae* subsp. rhinoscleromatis strain R-70 16S ribosomal RNA gene | NR_037084.1 |
| *Escherichia coli* strain NBRC 102203 16S ribosomal RNA | NR_114042.1 |
| *Escherichia coli* strain JCM 1649 16S ribosomal RNA | NR_112558.1 |
| *Escherichia coli* strain U 5/41 16S ribosomal RNA | NR_024570.1 |

**Table S3:** *Klebsiella* ssp. used in ANI and dDDH analysis.

| Sample/Species | Accession numbers | Genome |
| --- | --- | --- |
| *Klebsiella quasivariicola* 08A119 | GCF_020525665.1 | Reference |
| *Klebsiella huaxiensis* WCHKl090001 | GCF_003261575.2 | Reference |
| *Klebsiella indica* TOUT106 | GCF_005860775.1 | Reference |
| *Klebsiella africana* 200023 | GCF_020526085.1 | Reference |
| *Klebsiella spallanzanii* SB3356 | GCF_901563875.1 | Reference |
| *Klebsiella grimontii* LH87-a | GCA_024224355.1 | Reference |
| *Klebsiella pasteurii* Sb-24 | GCF_018139045.1 | Reference |
| *Klebsiella quasipneumoniae* KqPF26 | GCA_016415705.1 | Reference |
| *Klebsiella variicola* LEMB11 | GCF_009648975.1 | Reference |
| *Klebsiella michiganensis* THO-011 | GCF_015139575.1 | Reference |
| *Klebsiella aerogenes* Ka37751 | GCF_007632255.1 | Reference |
| *Klebsiella oxytoca* FDAARGOS_500 | GCF_003812925.1 | Reference |
| *Klebsiella pneumoniae* HS11286 | GCF_000240185.1 | Reference |
| *Klebsiella sp.* FDAARGOS_511 | GCF_003812845.1 | Complete |
| *Klebsiella sp.* KP20-425-1 | GCF_025813515.1 | Complete |
| *Klebsiella sp.* KPN54798 | GCA_021184225.1 | Complete |
| *Klebsiella sp.* KPN54798 | GCA_018204675.1 | Complete |
| *Klebsiella sp.* P1954 | GCA_018199955.1 | Complete |

**Table S4:** *K. aerogenes* genomes used in REALPHY, OrthoVenn and BPGA analysis

| Sample/Species | Accession numbers | Genome |
| --- | --- | --- |
| *Klebsiella aerogenes* Ka37751 | GCA_007632255.1 | Reference |
| *Klebsiella aerogenes* G7 | GCA_001571545.2 | Complete |
| *Klebsiella aerogenes* S33_CRE34 | GCF_027595705.1 | Complete |
| *Klebsiella aerogenes* FDAARGOS_327 | GCA_003546885.1 | Complete |
| *Klebsiella aerogenes* NY1688 | GCA_022759585.1 | Complete |
| *Klebsiella aerogenes* FDAARGOS_152 | GCF_001559215.2 | Complete |
| *Klebsiella aerogenes* 722 | GCF_024917575.1 | Complete |
| *Klebsiella aerogenes* Y1 | GCF_009732835.1 | Complete |
| *Klebsiella aerogenes* FDAARGOS_641 | GCF_008693885.1 | Complete |
| *Klebsiella aerogenes* Y3 | GCF_009732815.1 | Complete |
| *Klebsiella aerogenes* Y6 | GCF_009732795.1 | Complete |
| *Klebsiella aerogenes* FDAARGOS_513 | GCF_003812185.1 | Complete |
| *Klebsiella aerogenes* FDAARGOS_363 | GCF_002591115.1 | Complete |
| *Klebsiella aerogenes* 18-2341 | GCF_011067245.1 | Complete |
| *Klebsiella aerogenes* KAE3SP | GCF_019931695.1 | Complete |
| *Klebsiella aerogenes* FDAARGOS_139 | GCF_001593585.2 | Complete |
| *Klebsiella aerogenes* TS2020 | GCF_024661995.1 | Complete |
| *Klebsiella aerogenes* S2_CRE2 | GCF_027595505.1 | Complete |
| *Klebsiella aerogenes* NY1464 | GCF_022759605.1 | Complete |
| *Klebsiella aerogenes* CAV1320 | GCF_001021995.1 | Complete |
| *Klebsiella aerogenes* MINF_10B-sc-2280448 | GCF_904863085.1 | Complete |
| *Klebsiella aerogenes* EA46506 | GCF_016939495.1 | Complete |
| *Klebsiella aerogenes* AUH-KAM-9 | GCF_010509815.1 | Complete |
| *Klebsiella aerogenes* 57 | GCF_905333855.2 | Complete |
| *Klebsiella aerogenes* C9 | GCF_008931665.1 | Complete |
| *Klebsiella aerogenes* N1 | GCA_009937725.1 | Complete |

**Table S5:** Whole-Genome assembly and annotation statistics.

| **Content** | **Value** |
| --- | --- |
| **Total sequence lenght (bp)** | 5,462,831 |
| **Gene number** | 5,374 |
| **Gene lenght** | 4,855,563 |
| **GC content (%)** | 54,8 |
| **Gene lenght/Genome (%)** | 88,88 |
| **Gene average lenght** | 903,528 |
| **Intergenetic region length (bp)** | 607,268 |
| **Number of contigs** | 51 |
| **Contig N50 (bp)** | 263,170 |
| **Contig L50** | 7 |
| **Largest contig (bp)** | 546,266 |
| **Number of Coding Sequence** | 5,403 |
| **Number of rRNA** | 5 |
| **Number of tRNA** | 59 |
| **Number of tmRNA** | 1 |
| **Number of Subsystems** | 398 |

**
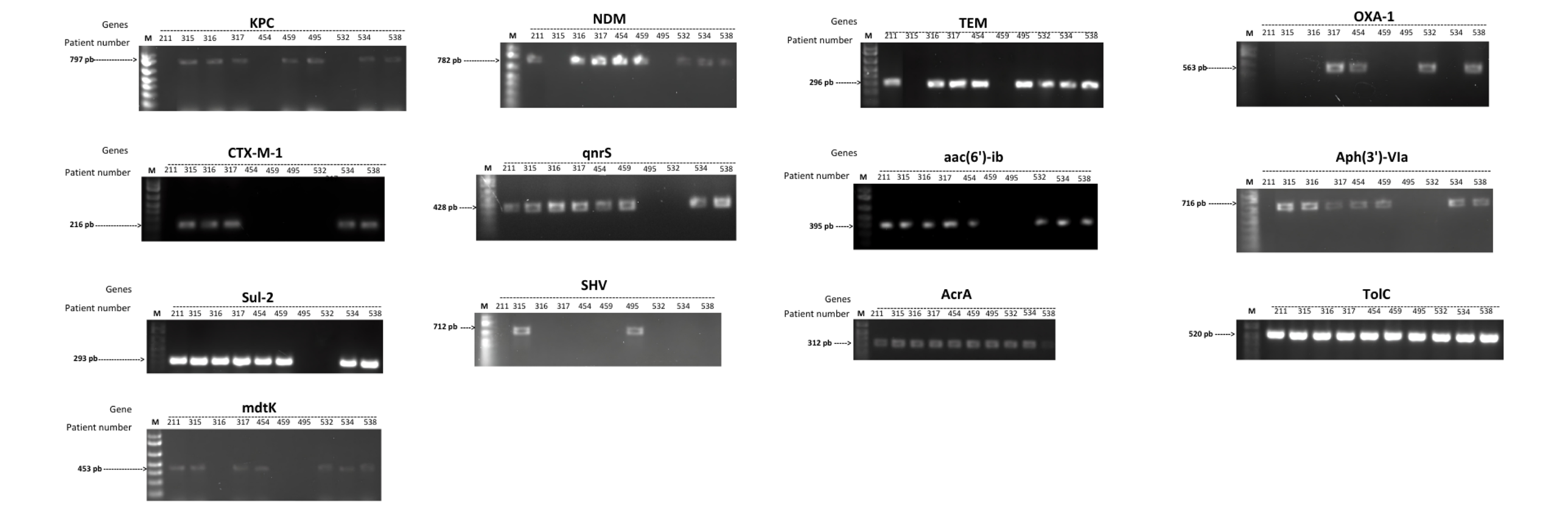
Figure S1: PCR was performed to amplify genes associated with resistance and efflux pumps in strains of *Klebsiella aerogenes*.** The amplified products were visualized on a 1% agarose gel stained with Ethidium bromide. A 1-kb DNA ladder was used as the size marker for comparison. Each lane in the electrophoresis gel corresponds to a specific patient's identification number.



**Figure S2. Mutations detected in the *mar*A and *sox*S genes of the *K. aerogenes* CRKA317.** (**A**) The amino acid sequence alignment. (B) One known mutation (Ser3Asn), two unknown mutations (Val96Ile, Gly103Glu) in multiple antibiotic resistance repressor (MarR), and unknown mutation in the gene SoxS (Ala12Glu). NA, not available.


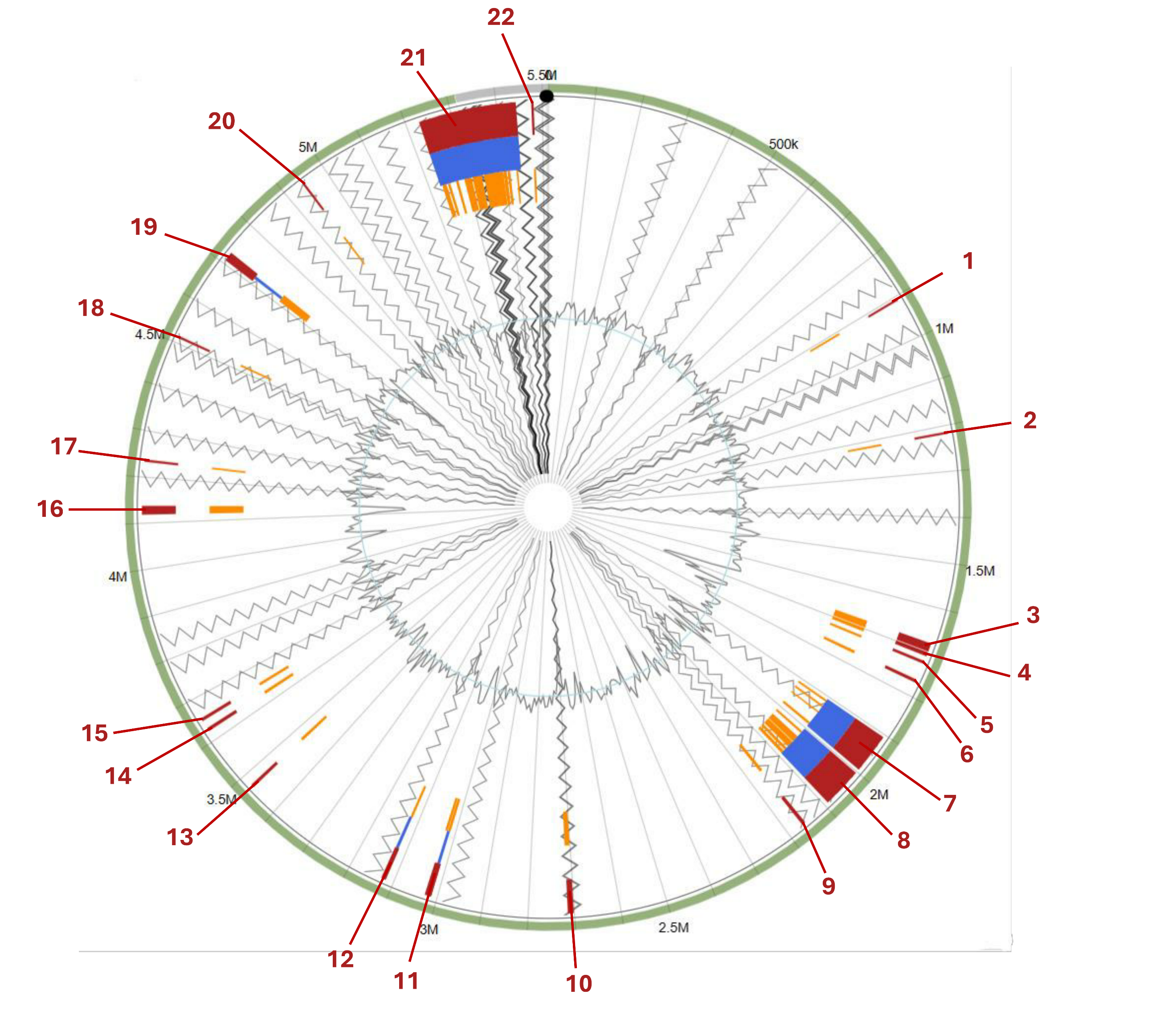


**Figure S3:** Circular representation of Genomic Islands in *K. aerogenes* CRKA317 genome. The green part of the circle represents aligned contigs and the gray part represents unaligned contigs. The red, blue and orange part represents islands that were predicted thought different methods (At least one method, IslandPath-DIMOB and SIGI-HMM, respectively).


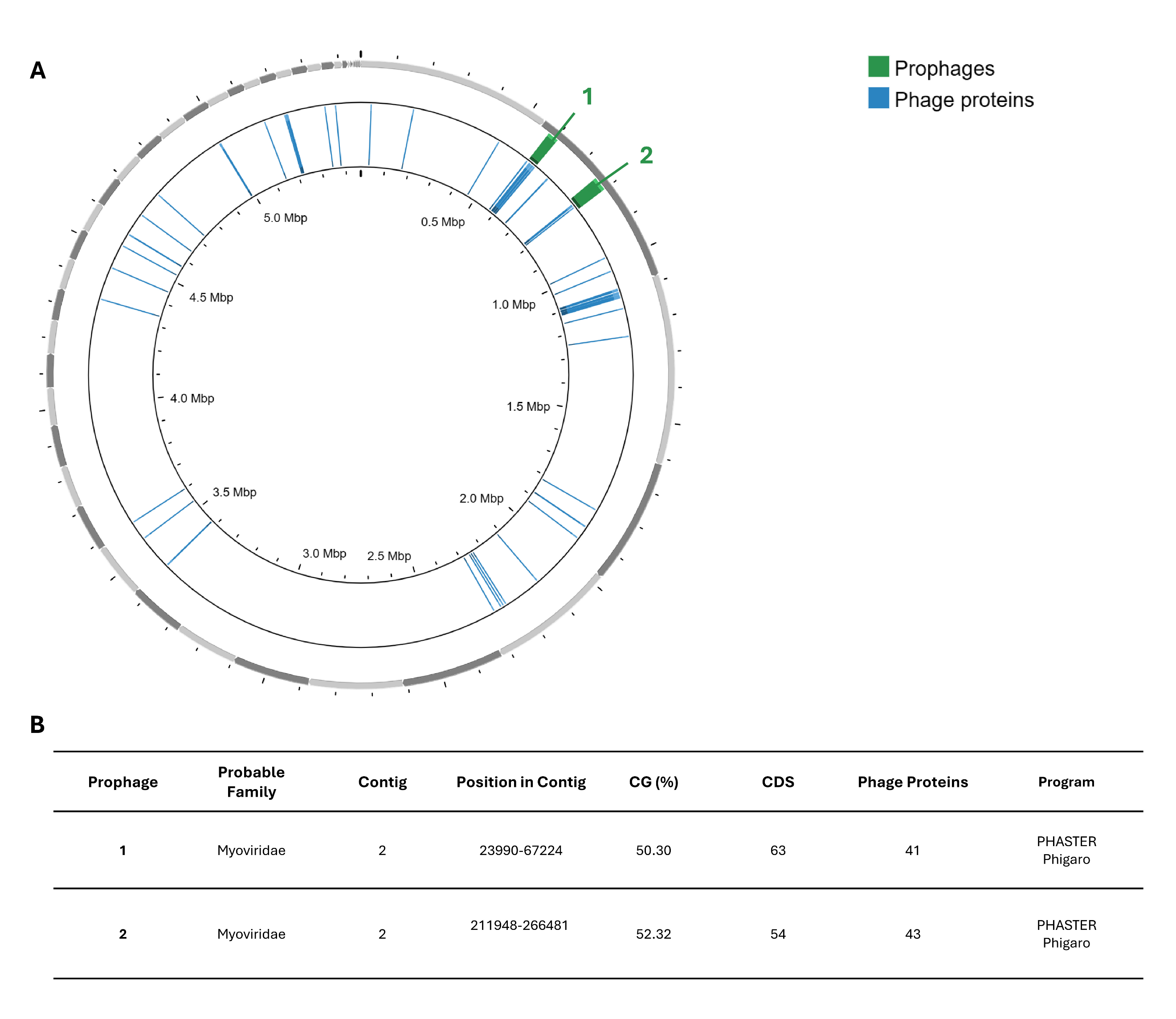
**Figura S4: Prophage componets in CRKA317 genome. (A)** Circular representation of the genome including phage proteins and prophage regions. **(B)** Description of prophage regions.

**REFERENCES**

Call, D. R., Bakko, M. K., Krug, M. J., and Roberts, M. C. (2003). Identifying Antimicrobial Resistance Genes with DNA Microarrays. *Antimicrob Agents Chemother* 47, 3290–3295. doi: 10.1128/AAC.47.10.3290-3295.2003.

Cattoir, V., Poirel, L., Rotimi, V., Soussy, C.-J., and Nordmann, P. (2007). Multiplex PCR for detection of plasmid-mediated quinolone resistance qnr genes in ESBL-producing enterobacterial isolates. *Journal of Antimicrobial Chemotherapy* 60, 394–397. doi: 10.1093/jac/dkm204.

Dallenne, C., Da Costa, A., Decré, D., Favier, C., and Arlet, G. (2010). Development of a set of multiplex PCR assays for the detection of genes encoding important β-lactamases in Enterobacteriaceae. *Journal of Antimicrobial Chemotherapy* 65, 490–495. doi: 10.1093/jac/dkp498.

Doyle, D., Peirano, G., Lascols, C., Lloyd, T., Church, D. L., and Pitout, J. D. D. (2012). Laboratory Detection of Enterobacteriaceae That Produce Carbapenemases. *J Clin Microbiol* 50, 3877–3880. doi: 10.1128/JCM.02117-12.

Ellington, M. J., Kistler, J., Livermore, D. M., and Woodford, N. (2006). Multiplex PCR for rapid detection of genes encoding acquired metallo- -lactamases. *Journal of Antimicrobial Chemotherapy* 59, 321–322. doi: 10.1093/jac/dkl481.

Jinwei Huang (2012). Coexistence of armA and genes encoding aminoglycoside-modifying enzymes in Acinetobacter baumannii. *Afr J Microbiol Res* 6. doi: 10.5897/AJMR11.1529.

Kerrn, M. B. (2002). Susceptibility of Danish Escherichia coli strains isolated from urinary tract infections and bacteraemia, and distribution of sul genes conferring sulphonamide resistance. *Journal of Antimicrobial Chemotherapy* 50, 513–516. doi: 10.1093/jac/dkf164.

Liu, Y.-Y., Wang, Y., Walsh, T. R., Yi, L.-X., Zhang, R., Spencer, J., et al. (2016). Emergence of plasmid-mediated colistin resistance mechanism MCR-1 in animals and human beings in China: a microbiological and molecular biological study. *Lancet Infect Dis* 16, 161–168. doi: 10.1016/S1473-3099(15)00424-7.

Maneewannakul, K., and Levy, S. B. (1996). Identification for mar mutants among quinolone-resistant clinical isolates of Escherichia coli. *Antimicrob Agents Chemother* 40, 1695–1698. doi: 10.1128/AAC.40.7.1695.

Martins, A. F., Zavascki, A. P., Gaspareto, P. B., and Barth, A. L. (2007). Dissemination of Pseudomonas aeruginosa Producing SPM-1-like and IMP-1-like Metallo-β-lactamases in Hospitals from Southern Brazil. *Infection* 35, 457–460. doi: 10.1007/s15010-007-6289-3.

Noppe-Leclercq, I., Wallet, F., Haentjens, S., Courcol, R., and Simonet, M. (1999). PCR detection of aminoglycoside resistance genes: a rapid molecular typing method for. *Res Microbiol* 150, 317–322. doi: 10.1016/S0923-2508(99)80057-6.

Platell, J. L., Cobbold, R. N., Johnson, J. R., Heisig, A., Heisig, P., Clabots, C., et al. (2011). Commonality among Fluoroquinolone-Resistant Sequence Type ST131 Extraintestinal *Escherichia coli* Isolates from Humans and Companion Animals in Australia. *Antimicrob Agents Chemother* 55, 3782–3787. doi: 10.1128/AAC.00306-11.

Poirel, L., Naas, T., and Nordmann, P. (2008). Genetic support of extended-spectrum β-lactamases. *Clinical Microbiology and Infection* 14, 75–81. doi: 10.1111/j.1469-0691.2007.01865.x.

Sheikhalizadeh, V., Hasani, A., Ahangarzadeh Rezaee, M., Rahmati-yamchi, M., Hasani, A., Ghotaslou, R., et al. (2017). Comprehensive study to investigate the role of various aminoglycoside resistance mechanisms in clinical isolates of Acinetobacter baumannii. *Journal of Infection and Chemotherapy* 23, 74–79. doi: 10.1016/j.jiac.2016.09.012.

Wasfi, R., Elkhatib, W. F., and Ashour, H. M. (2016). Molecular typing and virulence analysis of multidrug resistant Klebsiella pneumoniae clinical isolates recovered from Egyptian hospitals. *Sci Rep* 6, 38929. doi: 10.1038/srep38929.

Xiong, Z., Li, T., Xu, Y., and Li, J. (2007). Detection of CTX-M-14 extended-spectrum β-lactamase in Shigella sonnei isolates from China. *Journal of Infection* 55, e125–e128. doi: 10.1016/j.jinf.2007.07.017.

Xiong, Z., Zhu, D., Wang, F., Zhang, Y., Okamoto, R., and Inoue, M. (2004). A Klebsiella pneumoniae producing three kinds of class A β-lactamases encoded by one single plasmid isolated from a patient in Huashan Hospital, Shanghai, China. *Int J Antimicrob Agents* 23, 262–267. doi: 10.1016/j.ijantimicag.2003.07.011.

Yan, J.-J., Hsueh, P.-R., Ko, W.-C., Luh, K.-T., Tsai, S.-H., Wu, H.-M., et al. (2001). Metallo-β-Lactamases in Clinical Pseudomonas Isolates in Taiwan and Identification of VIM-3, a Novel Variant of the VIM-2 Enzyme. *Antimicrob Agents Chemother* 45, 2224–2228. doi: 10.1128/AAC.45.8.2224-2228.2001.

Yan, J.-J., Wu, J.-J., Ko, W.-C., Tsai, S.-H., Chuang, C.-L., Wu, H.-M., et al. (2004). Plasmid-mediated 16S rRNA methylases conferring high-level aminoglycoside resistance in Escherichia coli and Klebsiella pneumoniae isolates from two Taiwanese hospitals. *Journal of Antimicrobial Chemotherapy* 54, 1007–1012. doi: 10.1093/jac/dkh455.
